# Application of Digital Health Technologies in Scoliosis Rehabilitation: Systematic Review Based on the Technology Classification Framework

**DOI:** 10.2196/91461

**Published:** 2026-08-28

**Authors:** Yujie Guan, Jiaben Xu, Zelin Liu, Qi Qin, Xian Wang, Chengyuan An, Gege Wang, Zhiyuan Zhang, Maomao Gong, Bin Zhao

**Affiliations:** 1 Second Clinical Medical College, Heilongjiang University of Chinese Medicine, Harbin, Heilongjiang, China; 2 Department of Tuina, The Second Affiliated Hospital of Heilongjiang University of Chinese Medicine, Harbin, Heilongjiang, China; 3 Department of Orthopedics, The Second Affiliated Hospital of Heilongjiang University of Chinese Medicine, Harbin, Heilongjiang, China; 4 Department of Traditional Chinese Medicine, Harbin Beixiu Traditional Chinese Medicine Hospital, Harbin, Heilongjiang, China; 5 Department of Musculoskeletal Pain, The Second Affiliated Hospital of Heilongjiang University of Chinese Medicine, No. 411 Guogeli Street, Nangang District, Harbin, Heilongjiang, 150001, China, 86 18646592888

**Keywords:** scoliosis, digital health, physical therapy, telerehabilitation, virtual reality, wearable technology, systematic review

## Abstract

**Background:**

Digital health technologies are increasingly being used in scoliosis rehabilitation, but current evidence remains fragmented, and the effectiveness of these technologies across different categories, and their implementation in the real world remains unclear.

**Objective:**

We systematically evaluated the efficacy, implementation factors, and potential mechanisms of action of digital health technologies across various categories for scoliosis rehabilitation.

**Methods:**

We searched PubMed, IEEE Xplore, Embase, and Web of Science from inception to November 7, 2025, with an updated search on May 12, 2026. We included English-language controlled trials, cohort studies, and feasibility studies of digital interventions for any type of scoliosis that reported at least one quantitative outcome; nonoriginal publications, purely surgical studies, and those without extractable data were excluded. Risk of bias was assessed using the Cochrane Risk of Bias 2 tool (RoB 2) and the Risk of Bias in Nonrandomized Studies of Interventions (ROBINS-I), and intervention reporting completeness was assessed using the Template for Intervention Description and Replication (TIDieR). Interventions were categorized into 5 technology types and further stratified by evidence maturity into 3 tiers. Evidence was synthesized using vote counting based on the direction of effect, with prespecified subgroup and sensitivity analyses, and certainty of evidence assessed using the Grading of Recommendations Assessment, Development, and Evaluation (GRADE).

**Results:**

Thirteen studies (574 patients) from 5 countries were included. Among the randomized trials, 14.3% (n=1) were at high risk of bias, and among the nonrandomized studies, 33.3% (n=2) were at serious risk of bias. Across the 4 outcome domains, clinical outcomes showed a generally favorable direction for Cobb angle and flexibility but inconsistent evidence for the angle of trunk rotation (ATR); functional outcomes were consistently favorable for respiratory function and postural control; patient-reported quality-of-life outcomes were predominantly favorable, whereas evidence for pain and body image was inconsistent; implementation outcomes showed the least favorable overall direction of effect, with adherence and dropout more closely tied to supervision intensity than technology category. The main findings remained robust in sensitivity analyses; no serious adverse events were reported, and the overall certainty of evidence was low.

**Conclusions:**

Digital health interventions showed a generally favorable direction of effect for spinal alignment, function, and quality of life; however, evidence certainty was low, and outcomes should be interpreted with caution. To our knowledge, this is the first review to integrate diverse digital interventions into a single framework of 5 technology categories and 3 maturity tiers and to synthesize effectiveness across technologies, unlike prior single-technology or single-function reviews. Its main contribution is to shift the focus from technology effectiveness toward sustained use and to identify human supervision as a key factor influencing implementation. Clinically, technologies may be selected by maturity tier and paired with an appropriate supervision strategy to extend access to rehabilitation. Larger, long-term multicenter trials are needed for confirmation.

## Introduction

Scoliosis is a complex 3D spinal deformity characterized by lateral curvature in the coronal plane, deviations in the sagittal plane, and rotation in the axial plane [1-3]. Scoliosis is defined as a structural lateral curvature of the spine with a coronal Cobb angle ≥10° on a standing full-spine radiograph, typically accompanied by vertebral rotation [4]. It affects individuals across all age groups, from childhood to adulthood, and can be categorized into idiopathic scoliosis, congenital scoliosis, neuromuscular scoliosis, and syndromic scoliosis [5,6]. Scoliosis may cause postural abnormalities, low back pain, psychological issues, and, in severe cases, may lead to respiratory distress and diminished cardiopulmonary function [7]. Traditional conservative treatments primarily include bracing and physiotherapeutic scoliosis-specific exercises (PSSE) [8-10]. Although these methods have proven effective in controlling progression, clinical practice faces numerous challenges, including suboptimal patient adherence, insufficient continuous supervision during the rehabilitation process, limited access to traditional in-person rehabilitation, and difficulty in providing personalized real-time feedback, all of which constrain optimal treatment outcomes [11,12].

In recent years, the rapid advancement of digital health technologies has introduced novel solutions for the conservative management of scoliosis. Digital interventions such as telerehabilitation, virtual reality (VR), augmented reality (AR), wearable sensors, mobile health apps, and motion-based gaming are progressively being integrated into clinical practice [13-15]. Through real-time visual feedback, motion capture, remote supervision, and intelligent assessment, these technologies may enhance patients’ postural awareness, support treatment adherence, enable more precise training guidance, and help overcome geographical limitations to provide continuous rehabilitation support [16]. Existing research indicates that digital interventions demonstrate potential advantages in improving the Cobb angle, the angle of trunk rotation (ATR), postural symmetry, and patients’ quality of life. Furthermore, these interventions helped maintain the continuity of rehabilitation services during the COVID-19 pandemic [17,18].

Although digital interventions hold great promise for scoliosis rehabilitation, most primary studies in this field are small-scale and exploratory, and existing reviews vary in focus and have limited scope. Li et al [19] conducted a scoping review that systematically summarized the behavior change techniques (BCTs) and their theoretical mechanisms applied in digital exercise interventions for scoliosis; however, given the methodological scope of that design, clinical efficacy was not synthesized. Other relevant reviews have been limited to a single technology category or application domain, such as wearable devices in scoliosis management [20] or mobile apps for brace-wearing adherence monitoring [21]. To date, no review has evaluated the clinical effectiveness of digital interventions for scoliosis across technology categories, incorporated these technologies into a unified classification framework, or systematically analyzed implementation factors, such as supervision models and treatment adherence, that determine whether such benefits can be realized in real-world practice. Given that such technologies are rapidly entering routine scoliosis care and that clinical evidence to guide their selection and application remains lacking, addressing this gap in a timely manner holds significant practical importance.

To address these gaps, this review develops a technology classification framework for digital interventions in scoliosis rehabilitation, comprising 5 categories based on technical characteristics and 3 tiers based on evidence maturity, and uses this framework to organize the synthesis and comparison of evidence. Within this framework, the primary objective of this review is to systematically evaluate the clinical effectiveness of digital interventions for scoliosis rehabilitation across technology categories. The secondary objectives are 2-fold. First, to identify the key implementation factors, with a focus on supervision models and treatment adherence, that determine whether the observed efficacy translates into real-world benefits; and second, to explore the potential behavioral and neurophysiological mechanisms of digital interventions based on existing theoretical frameworks. This review aims to provide evidence for clinical decision-making and rehabilitation practice, thereby promoting the development and application of digital health technologies in standardized scoliosis treatment.

## Methods

### Overview

This systematic review was preregistered with the PROSPERO (International Prospective Register of Systematic Reviews; registration number: CRD420251250074) and was conducted and reported in accordance with the PRISMA (Preferred Reporting Items for Systematic Reviews and Meta-Analyses) 2020 statement (Checklist 1) [22]. The literature search process was reported in accordance with the PRISMA-S (PRISMA literature search extension) [23] to ensure full transparency (Checklist 2). Evidence synthesis was conducted in accordance with the Synthesis Without Meta-analysis (SWiM) reporting guideline [24] (Checklist 3).

Compared to the preregistered protocol, the following two protocol deviations were introduced during the study implementation: (1) search update: to ensure currency of the evidence base, a supplementary search was conducted after the initial search cutoff date to capture relevant literature published thereafter, and (2) adjustment of evidence synthesis strategy: the synthesis approach was adjusted from the originally planned narrative synthesis to a structured vote-counting method based on direction of effect, with predefined subgroup and sensitivity analyses, to enhance methodological rigor. Neither of these deviations affected the achievement of the study’s primary objectives.

### Eligibility Criteria

Eligibility criteria were predefined according to the PICOS (Population, Intervention, Comparator, Outcome, and Study design) framework.

Studies meeting all of the following criteria were included in this review: (1) P (population): patients diagnosed with any type of scoliosis, including adolescent idiopathic scoliosis (AIS), juvenile idiopathic scoliosis (JIS), adult degenerative scoliosis (ADS), and patients who have undergone posterior spinal fusion (PSF), regardless of age, sex, or curve severity; (2) I (intervention): any form of digital health intervention, including synchronous telerehabilitation, VR, AR, mobile health apps, wearable sensor systems, and digital health management platforms; (3) C (comparator): usual care, in-person rehabilitation, no active intervention, or other forms of digital intervention; (4) O (outcomes): at least one extractable quantitative outcome measure must be reported, covering radiographic parameters, quality of life, pain, respiratory function, postural parameters, assessment of surface deformity, adherence, or dropout rates; (5) S (study design): randomized controlled trials (RCTs), non-RCTs, cohort studies, and feasibility studies containing quantitative outcome data. Feasibility studies were included because they provide preliminary evidence of intervention feasibility, which is of particular value in a field where evidence remains limited.

Studies meeting any of the following criteria were excluded: (1) publications such as conference abstracts, case reports, narrative reviews, and study protocols that do not contain original quantitative data; (2) studies involving purely surgical interventions without a component of postoperative digital rehabilitation; (3) studies with insufficient raw data to support outcome extraction; and (4) non-English language literature.

### Information Sources

To ensure comprehensive coverage of biomedical, rehabilitation, and engineering literature, we systematically searched 4 electronic databases, each searched independently through its official platform (rather than using a unified, multidatabase search platform), including PubMed (MEDLINE), IEEE Xplore, Embase, and Web of Science Core Collection. The search covered both formally published and preprint literature, with no lower date limit, from the inception of each database to November 7, 2025. Additionally, citation searching was performed on the reference lists of all included studies to identify relevant studies that may have been missed in the database search. To systematically include literature published after the initial search cutoff date, an updated search of the aforementioned 4 databases was conducted on May 12, 2026, using the same search strategy and methods.

### Search Strategy

The search strategy combined MeSH terms with free-text keywords and was adapted to each database’s search guidelines to maximize search sensitivity. Core search terms covered 3 subject clusters: digital health interventions (eg, “digital health,” “telerehabilitation,” “virtual reality,” “mHealth,” “wearable,” and “exergame”), scoliosis (eg, “scoliosis,” “adolescent idiopathic scoliosis,” and “spinal curvature”), and therapeutic interventions (eg, “exercise,” “rehabilitation,” “Schroth,” “PSSE,” and “stabilization”). Searches were restricted to English-language publications. This decision was informed by methodological evidence indicating that language restrictions have a limited effect on the qualitative conclusions of systematic reviews of conventional medical interventions [25,26]; an English-language restriction is also a common practice, applied in approximately one-third of published systematic reviews [27]. No study design filters or published methodological search filters were applied during the search phase. The search strategy was developed by the research team based on the research questions, independently of previous systematic reviews, and was subsequently reviewed by an information specialist with expertise in systematic review searches to ensure its comprehensiveness and consistency. The full search strategies for all databases, including field tags and the number of records retrieved from each database, are reported in accordance with PRISMA-S [23] and provided in Multimedia Appendix 1.

The following search methods were outside the scope of the predefined search methodology and therefore were not implemented. This review did not search gray literature (including government reports, theses, or unpublished studies); clinical trial registries were not searched; no journal websites, institutional homepages, or conference proceedings were intentionally browsed; during data extraction, corresponding authors were contacted for clarification of unclear data, but field experts and manufacturers were not contacted for unpublished data; conference abstracts were explicitly excluded in the eligibility criteria, and conference proceedings databases were not searched separately; the search strategy was not formally peer-reviewed using the Peer Review of Electronic Search Strategies (PRESS) but was independently reviewed by an information specialist at our institution.

### Selection Process

The retrieved literature was imported into EndNote X9 (Clarivate) reference management software for automatic deduplication and manual verification. Two researchers (ZL and XW) independently screened all records based on titles and abstracts. Full-text assessment was conducted for records that passed the initial screening to confirm final eligibility, and reasons for exclusion were recorded for each record. Disagreements during the screening process were resolved through discussion and arbitrated by a third researcher (BZ). Interrater reliability for literature selection was Cohen κ=0.86. The complete literature selection process is presented in the PRISMA 2020 flow diagram.

### Data Collection Process

Two researchers (ZL and QQ) independently extracted data using a predesigned and pilot-tested standardized data extraction form. Disagreements were resolved through discussion, with a third researcher (BZ) serving as the arbitrator. Interrater reliability for data collection was Cohen κ=0.88. For missing or ambiguous data, the corresponding authors were contacted via email to seek clarification; if no response was received, the data were recorded as “not available.”

### Data Items

The data items extracted from each included study were predefined as follows: (1) basic study information: first author, year of publication, country, and study design; (2) participant characteristics: sample size, age, and type and severity of scoliosis; (3) intervention characteristics: type of digital technology, intervention content, supervision model, and intervention dose; (4) comparator group characteristics: comparator type and intervention content; (5) cointerventions: use of braces; (6) outcome data: measurement tools for each outcome measure, assessment time points, means and variability across groups, point estimates for between-group comparisons, *P* values, and effect sizes; (7) implementation: adherence and dropout and loss-to-follow-up rates. When multiple measurement time points exist for the same outcome measure, data at the end of the intervention were prioritized; when multiple measurement tools are used for the same outcome domain, data from each tool were extracted independently.

### Study Risk-of-Bias Assessment

Risk of bias in the included studies was assessed independently by 2 researchers (XW and CA). Disagreements were resolved through discussion, with a third researcher (MG) acting as an arbitrator when necessary. Interrater reliability for the risk of bias assessment was Cohen κ=0.88. For RCTs, the Cochrane Risk of Bias tool version 2 (RoB 2) [28] was used to assess 5 domains, including the randomization process, deviation from the intended intervention, missing outcome data, outcome measurement, and selective reporting. For nonrandomized studies, the Risk of Bias in Nonrandomized Studies of Interventions (ROBINS-I) tool [29] was used to assess 7 domains, including confounding, participant selection, classification of interventions, deviation from the intended intervention, missing data, outcome measurement, and selective reporting. Additionally, the Template for Intervention Description and Replication (TIDieR) checklist [30] was used to assess the completeness of intervention reporting across all included studies, focusing on the type of digital technology, intervention content, delivery method, supervision model, intervention frequency, duration, personalization strategies, and adherence monitoring methods.

The results of the risk-of-bias assessment were integrated into the evidence interpretation in 2 ways. First, by explicitly qualifying the conclusions of high-risk-of-bias studies in the outcome narrative and direction-of-effect synthesis; and second, by serving as the stratification basis for the prespecified sensitivity analyses.

### Synthesis Methods

This review did not conduct a quantitative meta-analysis for the following reasons: first, the included studies exhibited fundamental clinical differences in terms of technology type and core implementation mechanisms—including technical platforms, interaction modes, and intervention content—and forced pooling could result in unexplained mixed effects estimates. Second, the diversity of measurement tools within the same outcome domain limited direct comparability across studies. Third, for most outcomes, the number of studies eligible for pooling was too small to yield stable and reliable pooled effect estimates. Given these limitations, this review adopted an alternative synthesis strategy in accordance with the SWiM reporting guideline [24].

Following the procedures recommended by Boon and Thomson [31] and Chapter 12 of the Cochrane Handbook for Systematic Reviews of Interventions [32], evidence synthesis was performed using a vote-counting method based on the direction of effect. For each outcome, the direction of the point estimate from the between-group comparison served as the basic unit for synthesis, rather than statistical significance or effect sizes; this approach allowed studies with insufficient statistical power to be included in the synthesis based on their effect direction, rather than being excluded simply because they did not reach statistical significance [24,32]. Feasibility studies lacking a parallel control group were not included in the directional vote synthesis but were described qualitatively in a separate section to highlight their unique contributions. Effect direction coding followed 3 predefined categories, including favorable direction (↑), where point estimates indicate that the intervention group’s outcome is superior to that of the control group; unfavorable direction (↓), where point estimates indicate that the control group’s outcome is superior to that of the intervention group; and no clear direction (○), where point estimates are close to zero, suggesting that the outcomes of the 2 groups are substantially equivalent, or where the original study did not provide sufficient data to determine the direction of the between-group effect. When a single outcome domain included multiple measurement indicators, the directional coding was based on the predominant direction of each measure; if the directions of the measures were inconsistent and a predominant direction could not be determined, the coding was classified as no clear direction (○).

Based on the measurement characteristics and clinical significance of the outcomes, all outcome measures were categorized into four predefined domains: (1) clinical outcomes, reflecting the corrective effects of digital interventions on 3D spinal morphology; (2) functional outcomes, reflecting the impact of the intervention on patients’ physiological and motor functions; (3) patient-reported outcomes, reflecting patients’ subjective perceptions of health; and (4) implementation outcomes, reflecting the feasibility and sustainability of the intervention in real-world clinical settings.

For outcomes with at least 3 included studies, we conducted a prespecified 5-dimensional subgroup analysis to explore potential sources of heterogeneity in the direction of effects and their clinical significance: (1) technical pathway; (2) supervision model; (3) comparator type; (4) intervention dose; and (5) bracing cointervention status. The aforementioned subgroup analyses are exploratory in nature and aimed at identifying potential moderators of effect direction and were not designed to statistically test for moderating effects.

A sensitivity analysis was prespecified; the directional vote-counting synthesis was restricted to studies rated as low risk of bias or some concerns, excluding studies rated as high risk of bias by RoB 2 and those rated as serious risk of bias by ROBINS-I, to assess the extent to which the main synthesis conclusions depend on studies of lower methodological quality.

### Reporting Bias Assessment

This review did not conduct a meta-analysis and therefore did not produce pooled effect estimates. Statistical tests for publication bias based on pooled effect estimates (such as funnel plots and the Egger test) were not applicable to the synthesis framework of this study and were therefore not performed. The potential impact of not performing these assessments on the reliability of this review’s conclusions is discussed in the “Limitations” section.

### Certainty Assessment

The certainty of the evidence was assessed using the Grading of Recommendations Assessment, Development, and Evaluation (GRADE) approach. Given that this review used vote counting based on direction of effect and did not generate pooled effect estimates, certainty grading was conducted according to the method provided by Murad et al [33] for such syntheses, assessing 4 domains, including risk of bias, inconsistency, indirectness, and imprecision. The assessment was performed independently by 2 researchers (CA and GW); disagreements were resolved through discussion and, when necessary, arbitrated by a third researcher (MG).

## Results

### Study Selection

**Figure 1.**
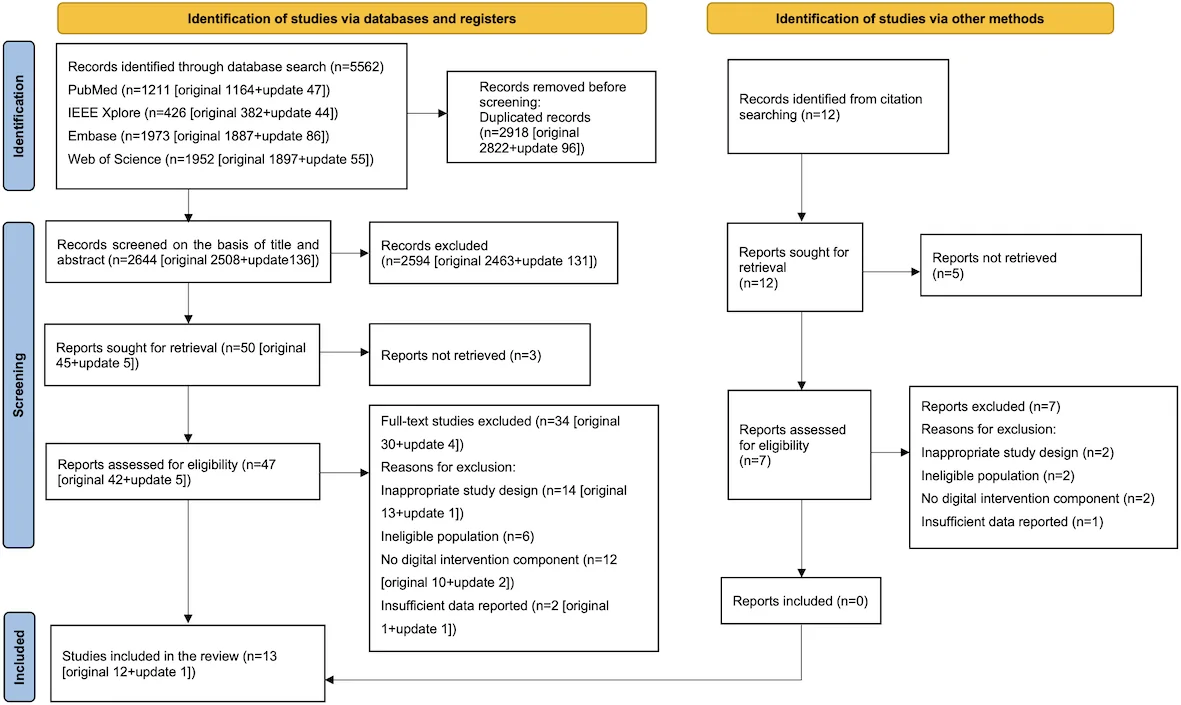
PRISMA (Preferred Reporting Items for Systematic Reviews and Meta-Analyses) 2020 flow diagram of the study selection process for digital health technologies in scoliosis rehabilitation.

A search of 4 databases identified a total of 5562 records. After deduplication, 2644 records were screened based on titles and abstracts; 2594 irrelevant records were excluded, leaving 50 records to proceed to the full-text retrieval stage. Of these, 3 studies were excluded due to unavailability of the full text, resulting in 47 studies undergoing full-text assessment. Subsequently, we excluded 34 studies that did not meet the eligibility criteria for study design (n=14), population (n=6), or intervention (n=12), or that had inadequate data reporting (n=2). Citation searching identified 12 additional records; of these, 5 were unavailable, and 7 were excluded after full-text assessment for failing to meet the inclusion criteria. Finally, 13 studies [17,34-45] were included in this review. The characteristics of the studies excluded after full-text assessment (n=41 [database search n=34 and citation searching n=7]) are detailed in Multimedia Appendix 2. The complete literature screening process is shown in Figure 1.

### Study Characteristics

The 13 included studies were published between 2015 and 2026, with study designs comprising 7 RCTs [35,37-40,43,44], 3 prospective studies [17,41,45], 1 non-RCT [36], and 2 feasibility studies [34,42]. Studies originated from 5 countries: China (n=4) [34,38,44,45], Turkey (n=4) [35,37,39,43], the United States (n=2) [36,40], Brazil (n=2) [17,41], and South Korea (n=1) [42]. The sample comprised 574 patients with various types of scoliosis: (1) AIS: 11 studies (84.6%) [17,35,36,38-45], involving 514 patients aged 9‐18 years; one study [40] included patients with AIS with concomitant low back pain, and another focused on patients after PSF [43]; (2) ADS: 1 study (7.7%) [34], involving 10 patients aged 53‐65 years; and (3) juvenile idiopathic arthritis (JIA) with scoliosis: 1 study (7.7%) [37], involving 50 patients aged 8‐16 years. Detailed characteristics of the included studies are summarized in Table 1.

**Table 1.** Characteristics of the 13 studies (7 RCTs, 3 prospective studies, 1 nonrandomized controlled trial, and 2 feasibility studies; 2015‐2026) included in this systematic review of digital health technologies for scoliosis rehabilitation.

Author	Year	Country	Study design	Sample size (T/C)^a^, n	Age (years)	Scoliosis type	Intervention group	Control group
Wan et al [34]	2024	China (Hong Kong)	Feasibility study (pre-post design)	10/0	53‐65	ADS^b^	Motion-sensing video game, exergame	—^c^
Andrade et al [17]	2024	Brazil	Prospective cohort study	33/33	10‐17	AIS^d^	Synchronous video-guided telerehabilitation + bracing	Traditional face-to-face rehabilitation + bracing
Dursun et al [35]	2024	Turkey	RCT^e^	16/15	10‐18	AIS	Initial clinic-supervised + subsequent synchronous video-guided hybrid telerehabilitation	Home-based unsupervised pilates exercise
Fishman [36]	2021	United States	Non-RCT	41/15	<21	AIS	Yoga isometric postures +partial remote video guidance	No intervention
Kisa et al [37]	2024	Turkey	RCT	25/25	8‐16	JIA^f^ with AIS	3D Schroth exercises + WhatsApp monitoring	Traditional core stability training + postural correction
Lau et al [38]	2021	China (Hong Kong)	RCT	20/20	11‐14	AIS	Mobile app+accelerometer+HIIT^g^ video	Usual care
Tombak et al [39]	2024	Turkey	RCT	19/18	10‐16	AIS	Supervised training + video-assisted review+3D scanning	Home training + video-assisted review+3D scanning
Zapata et al [40]	2015	United States	RCT	17/17	10‐17	AIS with low back pain	Supervised spinal stabilization training + DVD	Unsupervised spinal stabilization training + DVD
Moraes et al [41]	2022	Brazil	Prospective controlled study	7/7	11‐13	AIS	Immersive VR ^h^+ GPR^i^ postural training	GPR postural training
Nam et al [42]	2023	South Korea	Feasibility study (pre-post design)	13	—	AIS	AR^j^ smart barbell + mobile app guidance	—
Ozger et al [43]	2026	Turkey	RCT	14/14	—	Post-PSF^k^ patients	VR-assisted intelligent rehabilitation	Home exercise program
Yuan et al [44]	2025	China	RCT	64/64	9‐17	AIS	Digitalized system for remote supervision + PSSE^l^+health education	In-person supervised PSSE
Chen et al [45]	2026	China	Prospective cohort study	34/33	10‐15	AIS	Synchronous video tele-rehabilitation (Tencent Meeting) with real-time therapist-guided PSSE + CAD^m^-CAM^n^ Chêneau bracing	Guardian-supervised home-based PSSE with illustrated manual + CAD-CAM Chêneau bracing

a T/C: treatment/control group.

b ADS: adult degenerative scoliosis.

c Not available.

d AIS: adolescent idiopathic scoliosis.

e RCT: randomized controlled trial.

f JIA: juvenile idiopathic arthritis.

g HIIT: high-intensity interval training.

h VR: virtual reality.

i GPR: global postural reeducation.

j AR: augmented reality.

k PSF: posterior spinal fusion.

l PSSE: physiotherapeutic scoliosis-specific exercises.

m CAD: computer-aided design.

n CAM: computer-aided manufacturing.

### Risk of Bias in Studies

**Figure 2.**
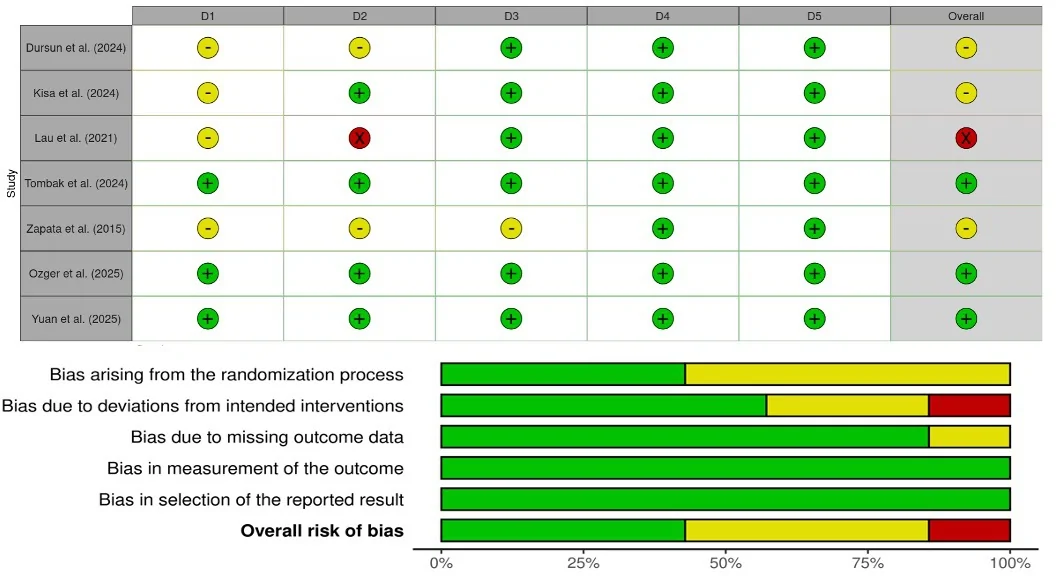
Risk-of-bias assessment for the 7 included randomized controlled trials with the RoB 2 [35,37-40,43,44].

**Figure 3.**
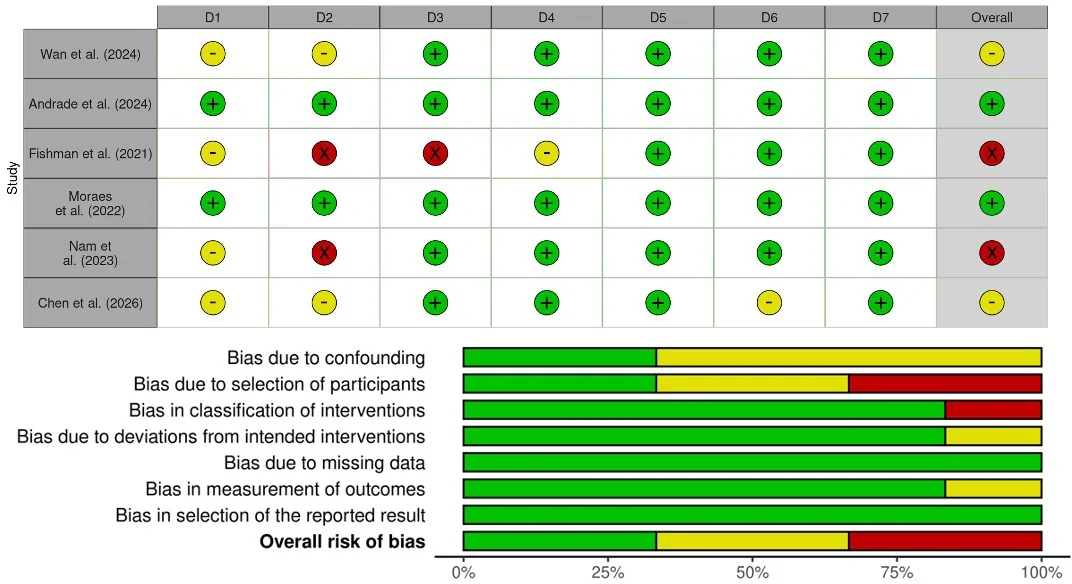
Risk-of-bias assessment for the 6 included nonrandomized studies with Risk of Bias in Nonrandomized Studies of Interventions (ROBINS-I) [17,34,36,41,42,45].

This systematic review used 3 complementary assessment tools to comprehensively evaluate the methodological quality of included studies and the completeness of intervention reporting. The RoB 2 assessment (Figure 2) indicated that 3 studies (42.9%) [39,43,44] had a low risk of bias, 3 studies (42.9%) [35,37,40] had some concerns, and 1 study (14.3%) [38] had a high risk of bias. Primary methodological limitations centered on 2 domains: the randomization process (unclear reporting of allocation concealment [35,37,38,40]) and deviation from the intended intervention (difficulty implementing participant and intervention provider blinding [35,38,40]). The ROBINS-I assessment (Figure 3) revealed 2 studies (33.3%) [17,41] were at low risk, 2 studies (33.3%) [34,45] were at moderate risk, and 2 studies (33.3%) [36,42] were at serious risk. Primary sources of bias included inadequate control of confounders [34,45], selection bias [34,36,42,45], and bias in the classification of interventions [36]. The TIDieR assessments (Table S1 in Multimedia Appendix 3) showed reporting completeness ranging from 75% to 100%. All studies adequately reported basic intervention characteristics (name, theoretical basis, materials, procedures, providers, and delivery methods), but reporting on fidelity monitoring and adherence assessment was relatively limited. The synthesis of results from 3 assessment tools indicated that included studies exhibited methodological heterogeneity, with RCTs demonstrating superior overall quality to that of nonrandomized studies. Given the exploratory nature of digital interventions in scoliosis rehabilitation and limited existing evidence, this review retained all included studies to provide a comprehensive evidence base. However, interpretation of findings must fully account for the methodological limitations of each study.

In Figure 2, the upper panel shows the domain-level and overall judgment for each trial, and the lower panel shows the distribution of judgments across trials. Domains are D1, bias arising from the randomization process; D2, bias due to deviations from intended interventions; D3, bias due to missing outcome data; D4, bias in measurement of the outcome; and D5, bias in selection of the reported result. Judgments are low risk (green plus sign), some concerns (yellow minus sign), and high risk (red cross).

In Figure 3, the upper panel shows the domain-level and overall judgment for each study, and the lower panel shows the distribution of judgments across studies. Domains are D1, bias due to confounding; D2, bias due to selection of participants; D3, bias in classification of interventions; D4, bias due to deviations from intended interventions; D5, bias due to missing data; D6, bias in measurement of outcomes; and D7, bias in selection of the reported result. Judgments are low risk (green plus sign), moderate risk (yellow minus sign), and serious risk (red cross).

### Digital Intervention Program

Detailed characteristics of the digital technologies included in the studies are presented in Table 2. Based on core technical features, the digital intervention programs included in the studies can be categorized into five primary technical pathways (Table 3): (1) synchronous telerehabilitation technology was applied in five studies [17,35-37,45], with 3 using generic instant messaging platforms (WhatsApp; Meta [17,37]; Tencent Meeting, Tencent [45]), one using a hybrid telerehabilitation model (initial in-person clinic sessions combined with subsequent remote monitoring) [35], and one using professional video conferencing platforms such as Skype (Microsoft Corp) and Zoom (Zoom Video Communications, Inc) [36]; (2) asynchronous digital educational tools were applied in 3 studies [38-40], including 2 prerecorded video studies ([40] using digital versatile discs; DVDs [39], using self-recorded videos) and 1 mobile app video library [38]; (3) immersive VR and AR technologies were applied in 4 studies [34,41-43], with 3 using VR gamified systems (Vicon system; Oxford Metrics Group, HTC Vive; HTC Corp, Kinect Xbox 360; Microsoft [34,41,43]) and 1 developing an AR smart-bar device integrated with a 9-axis inertial sensor and mobile app for real-time guidance [42]; (4) wearable biosensing technology was applied in 2 studies [38,42]. One study [38] used wrist-worn accelerometers to track high-intensity interval training (HIIT) activity levels, while another [42] used a 9-axis inertial sensor for real-time detection of postural deviations; (5) integrated digital health platforms were applied in 1 study [44], in which the authors developed an integrated digital health management platform combining patient-side apps, therapist management interfaces, and cloud-based data analysis to deliver personalized training plans based on the Peking Union Medical College Hospital Scoliosis-Specific Exercise (PUMCH-SSE) classification.

**Table 2.** Characteristics of the digital health interventions evaluated in the 13 included studies of scoliosis rehabilitation. Wan et al [34] and Nam et al [42] were feasibility studies; their reported outcomes were summarized qualitatively and were not entered into the direction-of-effect synthesis.

Author	Digital intervention protocol	Frequency	Advantages and features	Primary intervention outcomes
Wan et al [34]	Developed a motion-sensing video game using optical sensors (Vicon system) for real-time monitoring of sitting and standing postures. Players controlled game character speed and trajectory by adjusting posture, with real-time visual feedback.	3 sessions/week, 1 hour/session, for 6 weeks	Real-time visual feedback; adaptive difficulty adjustment; integrated optical motion capture and sEMG^a^ monitoring	Postural alignment; muscle symmetry
Andrade et al [17]	Synchronous telerehabilitation via WhatsApp video, with physical therapists providing real-time guidance for S4D^b^-specific exercises, including axial elongation, 3-plane postural correction, and breathing training, combined with 18‐20 hours daily bracing.	1 session/week, 60 minutes/session, for 6 months	Real-time bidirectional video supervision (WhatsApp); integrated with bracing and home exercise program	①^c^
Dursun et al [35]	Hospital-supervised Pilates training for the first 2 weeks, followed by 10 weeks of synchronous video conference-guided remote training by physical therapists. Patients performed self-directed exercises on remaining days.	1 hour/day, for 12 weeks	Hybrid clinic-plus-remote model; individualized exercise protocol; real-time audiovisual feedback; integrated breathing and postural correction components	①②④^d^^,^^e^
Fishman [36]	Remote guidance via video conferencing platforms (Skype, Zoom, FaceTime) for specific yoga postures (side plank, half-moon, lifted side plank). Postures were individualized based on scoliosis type, emphasizing convex side down to strengthen weaker muscle groups.	1 session/day, postures maintained as long as possible, for at least 5 months	Consumer video-conferencing platforms (Skype, Zoom, and FaceTime); no specialized equipment; curve-specific posture selection with convex-side-down loading	①⑨^f^
Kisa et al [37]	3D Schroth exercises with WhatsApp remote monitoring for home training guidance, supervision, and feedback.	First 6 weeks: 2 sessions/week (face-to-face rehabilitation); following 18 weeks: daily home training with WhatsApp remote monitoring	Daily WhatsApp-based training monitoring and feedback; integrated postural correction, breathing training, and core strengthening	①②④⑦⑧⑨⑩^g,h,i^
Lau et al [38]	Mobile app providing randomly generated 7-minute HIIT^j^ videos (40 types of high-impact weight-bearing exercises), with accelerometer monitoring of activity levels.	5 days/week, approximately 7 minutes/day, for 6 months	Short-duration high-intensity protocol (7 min/session); randomized video-sequence generation from a 40-exercise library; activity monitoring via mobile app and wrist accelerometer	①⑥⑨^k^
Tombak et al [39]	Schroth exercises with supervised group training at clinic + video review, home group viewing self-recorded videos + monthly follow-up visits, both groups using Artec Eva 3D scanner for assessment.	1 hour/day, for 12 weeks	3D surface scanning (Artec Eva) for assessment; self-recorded video review; monthly in-person reassessment	②⑥⑧⑨
Zapata et al [40]	DVD containing core muscle training including transversus abdominis and erector spinae exercises.	First 2 weeks: 5 days/week; following 6 weeks: 3 days/week; 20 minutes/session	Standardized DVD-based home protocol with written manual; core-stabilizer training (transversus abdominis and erector spinae)	⑦⑨⑩
Moraes et al [41]	Immersive VR^l^ system (HTC Vive headset + Wii Balance Board), ballet dancer postural training with real-time visual feedback and gamified rewards.	≥7-d intervals, 3‐4 sessions	Multimodal (visual and balance) biofeedback; gamified reward mechanism	③⑤^m^^,^^n^
Nam et al [42]	AR^o^ smart-bar device (9-axis sensors)+mobile app, real-time AR body frame overlay guidance with audio feedback for error correction.	—^p^	Nine-axis sensor-based motion-angle measurement; real-time AR body-frame overlay; auditory error-correction feedback	Movement accuracy; measurement validity
Ozger et al [43]	VR rehabilitation (Kinect Xbox 360 games) including football, track and field, boxing, volleyball, and other dynamic games involving trunk lateral flexion, extension, and upper limb movements.	2 sessions/week, 30 minutes/session, for 6 weeks	Kinect-based gamified sports exercises (football, boxing, volleyball, and track-and-field); whole-body dynamic training under therapist supervision	③④⑤⑥⑨
Yuan et al [44]	Digitalized system providing remote PSSE^q^ supervision, automated reminders, educational videos, and real-time therapist communication, with individualized training based on PUMCH-SSE^r^ classification.	Daily training, for 6 months	Integrated digital platform with PUMCH-SSE–based individualized plans; automated reminders; educational videos; bidirectional real-time therapist communication	①②⑤⑨⑩
Chen et al [45]	Synchronous tele-rehabilitation via Tencent Meeting, with physical therapists providing real-time visual supervision and corrective feedback for Schroth-based PSSE, combined with individualized CAD^s^-CAM^t^–fabricated Chêneau bracing (≥22 hours/day). Digital attendance tracking with automated reminders and an incentivization protocol (≥90% adherence rewarded with educational materials) were implemented.	5 sessions/week, 40 minutes/session, for 24 months	Real-time synchronous supervision with immediate kinematic error correction; integrated CAD-CAM Chêneau bracing; digital attendance tracking and automated reminders; incentivization protocol (≥90% adherence rewarded)	①②⑥⑨⑩

a sEMG: surface electromyography.

b S4D: Scientific Exercises Approach to Scoliosis.

c ①: Cobb angle.

d ②: angle of trunk rotation.

e ④: respiratory function.

f ⑨: adherence.

g ⑦: pain.

h ⑧: body image.

i ⑩: dropout and loss-to-follow-up rate.

j HIIT: high-intensity interval training.

k ⑥: health-related quality of life.

l VR: virtual reality.

m ③: spinal flexibility.

n ⑤: postural control and gait.

o AR: augmented reality.

p Not applicable.

q PSSE: physiotherapeutic scoliosis-specific exercises.

r PUMCH-SSE: Peking Union Medical College Hospital Scoliosis-Specific Exercise.

s CAD: computer-aided design.

t CAM: computer-aided manufacturing.

**Table 3.** Technology classification and clinical application characteristics of the included digital health interventions across 5 technical pathways.

Technology category	Number of included studies	Core technology components and implementation methods	Feedback mechanism
Synchronous telerehabilitation technologies	5	—^a^	—
Generic instant messaging platforms	3 [17,37,45]	Consumer-grade video calling software (eg, WhatsApp [Meta], Tencent Meeting [Tencent]) with supplementary digital attendance tracking and automated adherence reminders	Visual+auditory
Hybrid telerehabilitation model	1 [35]	Initial clinic-based face-to-face instruction to establish foundation, followed by video conference supervision for quality maintenance	Visual+auditory+tactile
Professional telerehabilitation platforms	1 [36]	Customized video conferencing system integrated with patient management functions	Visual+auditory+data tracking
Asynchronous digital educational tools	3	—	—
DVD and prerecorded videos	2 [39,40]	Physical media or downloadable video files	Visual+auditory
Mobile app video libraries	1 [38]	Streaming educational videos and articles integrated within app	Visual+auditory+text
Immersive VR^b^ and AR^c^ technologies	4	—	—
VR gamified systems	3 [34,41,43]	Head-mounted displays or optical tracking+motion sensing control using HTC Vive, Kinect Xbox, and Vicon systems	Visual+auditory+haptic vibration
AR motion guidance systems	1 [42]	Smart-bar device + 9-axis sensors + mobile app with real-time AR body frame overlay	Visual+auditory+tactile+virtual imagery
Wearable biosensing technologies	2	—	—
Accelerometers and pedometers	1 [38]	Wrist-worn accelerometer sensors for tracking HIIT^d^ training activity levels	Visual
Intelligent posture monitoring systems	1 [42]	Multiaxis inertial sensors (9-axis IMU^e^) for real-time detection of body posture deviation	Visual+auditory alerts+tactile
Integrated digital health platforms	1	—	—
Digital health care integrated systems	1 [44]	Patient-side app+therapist management portal+cloud-based data analytics, with personalization based on PUMCH-SSE^f^ classification	Visual+auditory+data+automated reminders

a Not applicable.

b VR: virtual reality.

c AR: augmented reality.

d HIIT: high-intensity interval training.

e IMU: inertial measurement unit.

f PUMCH-SSE: Peking Union Medical College Hospital Scoliosis-Specific Exercise.

The interventions included in the studies exhibited considerable heterogeneity in frequency, duration, supervision model, and exercise methods. Regarding intervention frequency: low frequency (≤2 sessions/week) was used in 3 studies [17,37,43]; moderate frequency (3‐5 sessions/week) in 2 studies [34,40]; high frequency (6‐7 sessions/week) in 6 studies [35,36,38,39,44,45]; 1 study [41] used an intermittent frequency (≥7 day intervals, totaling 3‐4 sessions); and 1 study [42] did not report frequency. Regarding intervention duration, 6 studies [17,36-38,44,45] delivered long-term interventions (total duration >12 weeks), 6 [34,35,39-41,43] delivered short-term interventions (≤12 weeks), and Nam et al [42] did not report a standardized duration.

Based on the mode and intensity of therapist involvement during the intervention, supervision models can be classified into three categories: (1) real-time synchronous therapist supervision (6 studies [17,34,35,41,43,45]): during each training session, therapists actively observed and corrected movement execution through in-person guidance [34,41,43] or real-time video links [17,35,45]; (2) asynchronous technology-mediated remote monitoring (3 studies [37,38,44]): patients primarily engaged in independent home-based practice, with delayed or on-demand therapist feedback facilitated through instant messaging [37], mobile apps, and wearable device tracking [38], or digital health management platforms [44]; no synchronous supervision occurred during training; (3) minimally supervised self-directed practice (4 studies [36,39,40,42]): following initial instruction, training was primarily self-directed at home, with professional contact limited to occasional remote guidance [36], video review combined with periodic follow-ups [39], or the provision of standardized video instructional materials only [40]; there was no continuous real-time or technology-mediated monitoring.

Among the 11 studies with parallel control groups, the control groups can be classified into three categories based on increasing intervention intensity: (1) inactive comparator (2 studies [36,38]): The control group received usual care or no active intervention; (2) unsupervised home exercise (3 studies [35,43,45]): the control group performed home exercises without real-time supervision by a professional; (3) in-person supervised rehabilitation (6 studies [17,37,39-41,44]): the control group received traditional rehabilitation training under the in-person guidance of a professional therapist. Two feasibility studies [34,42] were not included in the above classification because they lacked parallel control groups.

Exercise methods exhibited diverse characteristics. Four studies [37,39,44,45] applied 3D corrective training based on the PSSE method, emphasizing self-corrective posture, rotational breathing, and spinal stability training. One study used a modified Pilates protocol [35], focusing on core muscle strengthening and breathing exercises. Another study used yoga isometric posture training [36], incorporating poses such as side plank and half moon. Four studies [34,41-43] integrated motion-sensing games or VR (AR) systems, combining postural control with movement feedback. One study used HIIT training [38], incorporating 40 high-impact weight-bearing exercises. Additionally, 3 studies combined orthotic treatment; all patients in Andrade et al [17] wore thoracolumbosacral orthoses for 18‐20 hours/day; 50% of participants in Dursun et al [35] used orthoses; and all participants in Chen et al [45] received an individualized computer-aided design and computer-aided manufacturing (CAD-CAM)–fabricated Chêneau brace, worn ≥22 hours/day.

### Stratified Reporting of Outcome Measures

This section presents stratified reports according to the 4 outcome domains defined in the methodology (clinical outcomes, functional outcomes, patient-reported outcomes, and implementation outcomes). For studies with a parallel control group, results were synthesized using a vote-counting method based on the direction of effect; 2 feasibility studies were excluded from the direction-of-effect synthesis due to the absence of a parallel control group, and their outcomes were described qualitatively. The complete extracted outcome data for each included study are presented in Multimedia Appendix 4.

### Clinical Outcomes

#### Clinical Outcomes Overview

Clinical outcomes encompass 3 measures, namely Cobb angle, ATR, and spinal flexibility. Based on a direction-of-effect summary from 11 nonfeasibility studies [17,35-41,43-45] (Table S1 in Multimedia Appendix 5), the proportions of studies showing a favorable direction for the 3 measures were as follows: Cobb angle (n=5, 71.4%), ATR (n=2, 40.0%), and spinal flexibility (n=2, 100%). The Cobb angle and spinal flexibility showed a relatively consistent favorable direction, whereas the proportion with a favorable direction for ATR was relatively low, suggesting uncertainty regarding the efficacy of digital health interventions in improving spinal rotational deformities.

#### Cobb Angle

A total of 7 studies [17,35-38,44,45] reported on the Cobb angle; 5 [35-37,44,45] showed a favorable direction, one [17] showed an unfavorable direction, and one [38] showed no clear direction. Among the 5 studies showing a favorable direction, the strength of evidence varied. Three studies reported statistically significant between-group mean differences that exceeded the minimal clinically important difference (MCID) for Cobb angle (3.5°), as established by Schreiber et al [46] using an anchor-based method in an RCT of patients with AIS: Yuan et al [44] (between-group difference of 4.23°, *P*<.001), Kisa et al [37] (between-group difference of 4.00°, *P*<.001), and Dursun et al [35] (between-group difference of 4.04°, *P*=.01). Chen et al [45] reported a between-group difference of 3.30° (*P*=.02), which was statistically significant but below the MCID threshold. Fishman [36] reported that the intervention group’s thoracolumbar and thoracic Cobb angles decreased by 9.2° and 7.1°, respectively, while the control group’s angles worsened by 5.4° and 9.3°, respectively, during the same period; however, due to a serious risk of bias, the reliability of this evidence is limited. Andrade et al [17] was the only study showing an unfavorable direction. The telerehabilitation group showed a within-group improvement of 2.4° (*P*=.03; d=0.33), while the traditional in-person control group showed a within-group improvement of 4.9° (*P*=.02; d=0.74). The point estimate favored the control group; however, this between-group difference was not statistically significant, suggesting that synchronous telerehabilitation did not outperform in-person rehabilitation for Cobb angle improvement. Lau et al [38] conducted the only study with no clear direction; technical failure of the wrist-worn accelerometer resulted in a low synchronization rate (approximately 15%), so adherence could not be reliably confirmed and inadequate treatment exposure could not be excluded.

The 5-dimensions subgroup analysis (Tables S2-S6 in Multimedia Appendix 5) generally supports the main synthesis direction. The comparator-type dimension showed an interpretable pattern in the direction of effects; both studies using unsupervised home exercise as the control group showed a favorable direction [35,45]; among the 3 studies using in-person supervised rehabilitation as the control group, 2 showed a favorable direction [37,44] and one showed an unfavorable direction [17]; of the 2 studies using no intervention as a control, one showed a favorable direction [36] and one showed no clear direction [38]. This preliminarily suggests that the intensity of the comparator may be one of the moderating factors influencing the direction of effect for the Cobb angle; however, due to the limited number of studies in each subgroup, this is not yet sufficient evidence. No systematic directional deviation was observed in the remaining 4 dimensions.

#### ATR

A total of 5 studies reported on ATR [35,37,39,44,45]; 2 showed a favorable direction [37,45], 3 showed no clear direction [35,39,44], and none showed an unfavorable direction. Both studies showing a favorable direction reported statistically significant between-group differences. Kisa et al [37] reported a 4.1° improvement in ATR in the intervention group, compared to 2.2° in the control group (*P*=.007); Chen et al [45] reported that at the 24-month follow-up, the final ATR value in the telerehabilitation group (mean 6.9°, SD 1.9°) was superior to that in the control group (mean 8.7°, SD 2.9°; *P*=.005). Among the 3 studies with no clear direction, both groups in Dursun et al [35] and Yuan et al [44] showed improvements in ATR from baseline, but the differences between groups were not statistically significant; in Tombak et al [39], both groups achieved statistically significant within-group improvements, but the magnitude of improvement between groups was comparable.

In the 5-dimensions subgroup analysis (Tables S2-S6 in Multimedia Appendix 5), the intervention dose dimension showed patterns potentially related to the direction of effect. In the long-term intervention subgroup (total duration >12 weeks), 2 studies [37,45] showed a favorable direction and one [44] showed no clear direction; in the short-term intervention subgroup (≤12 weeks), both studies showed no clear direction [35,39]. This pattern preliminarily suggests that intervention duration may be one of the moderating factors influencing the direction of effect for ATR; however, due to the limited number of studies in each subgroup, further validation is required. No systematic directional deviation was observed in the remaining 4 dimensions.

#### Spinal Flexibility

A total of 2 studies reported on spinal flexibility [41,43], both of which showed a favorable direction, with no study showing an unfavorable direction or no clear direction. Ozger et al [43] reported that lumbar flexion range of motion in the VR-assisted rehabilitation group increased from mean 19.29 (SD 1.54) cm to mean 20.71 (SD 1.49) cm (within-group *P*<.001), while the control group showed no statistically significant change during the same period; the between-group difference reached statistical significance (*P*=.04). Moraes et al [41] also reported that immersive VR improved parameters related to spinal flexibility. Both studies used immersive VR and showed a consistent favorable direction for spinal flexibility. Given that only 2 studies reported this outcome, no subgroup direction-of-effect synthesis was conducted.

### Functional Outcomes

#### Functional Outcomes Overview

Functional outcomes encompass 2 measures: respiratory function and postural control and gait. Based on a direction-of-effect summary from 11 nonfeasibility studies [17,35-41,43-45] (Table S1 in Multimedia Appendix 5), the proportions of studies showing a favorable direction for the 2 measures were as follows: respiratory function (n=3, 100%) and postural control and gait (n=3, 100%), indicating a consistent favorable direction for both measures.

#### Respiratory Function

A total of 3 studies [35,37,43] reported on respiratory function, all of which showed a favorable direction and reported statistically significant between-group differences; no study showed an unfavorable direction or no clear direction. Dursun et al [35] reported that after a 12-week blended remote Pilates intervention, the intervention group showed greater increases in maximal inspiratory pressure (MIP; +25.43 cm H₂O), maximal expiratory pressure (MEP; +28.50 cm H₂O), and peak expiratory flow percent predicted (PEF%; +13.87%) compared to the control group (MIP: P=.01; MEP: P=.03; PEF%: P=.03). Kisa et al [37] reported that the 3D Schroth remote training group showed greater improvements in forced vital capacity percent predicted (FVC%; +6.36%) and forced expiratory volume in 1 second percent predicted (FEV1%; +7.84%) compared to the control group (*P*=.02; *P*=.04). Ozger et al [43] reported that the increase in MIP (+20.64 cm H₂O) in the VR-assisted rehabilitation group was higher than that in the control group (+5.21 cm H₂O; *P*=.02). Although the 3 studies differed in their assessment dimensions, the favorable direction was consistent across studies.

The 5-dimensions subgroup analysis (Tables S2-S6 in Multimedia Appendix 5) was consistent with the main synthesis, with all data subgroups showing a favorable direction and no systematic directional deviation observed. This preliminarily suggests that the favorable direction for respiratory function exhibits a certain degree of cross-subgroup stability within the scope of the current evidence.

#### Postural Control and Gait

A total of 3 studies [41,43,44] reported on postural control and gait, all of which showed a favorable direction, with no study showing an unfavorable direction or no clear direction. The strength of evidence varied among the 3 studies. Two of these studies reported statistically significant between-group differences. Yuan et al [44] reported that the digital intervention group had superior pelvic tilt angles compared to the control group during the 4 characteristic phases of the gait cycle (difference range 0.76°-1.23°; P=.02, P=.02, P=.02, and P=.04, respectively). Moraes et al [41] reported that the VR training group had a longer standing tolerance time than the control group (*P*=.001) and a smaller shift in the center of pressure during sitting than the control group (*P*=.002). Ozger et al [43] reported that the VR-assisted rehabilitation group achieved statistically significant within-group improvements in pelvic tilt, sagittal vertical axis (SVA), and Tampa Scale for Kinesiophobia (TSK) scores, while the control group showed no statistically significant changes during the same period; however, none of the between-group comparisons reached statistical significance. The direction was coded from the asymmetric within-group improvements between the 2 groups, and the strength of evidence was relatively weak.

The 5-dimensions subgroup analysis (Tables S2-S6 in Multimedia Appendix 5) was consistent with the main synthesis direction, with all data subgroups showing a favorable direction and no systematic directional deviation observed. This preliminarily suggests that the favorable direction for postural control and gait exhibits a certain degree of cross-subgroup stability within the scope of the current evidence.

### Patient-Reported Outcomes

#### Patient-Reported Outcomes Overview

Patient-reported outcomes encompass 3 measures: health-related quality of life, pain, and body image. Based on a direction-of-effect summary from 11 nonfeasibility studies [17,35-41,43-45] (Table S1 in Multimedia Appendix 5), the proportions of studies showing a favorable direction for the 3 measures were as follows: quality of life (n=3, 75.0%), pain (n=1, 50.0%), and body image (n=1, 50.0%). Quality of life showed a relatively consistent favorable direction; for pain, both favorable and unfavorable directions were observed, indicating conflicting directional evidence; for body image, no study showed an unfavorable direction, but the limited number of included studies restricts the robustness of the synthesis conclusions.

#### Health-Related Quality of Life

A total of 4 studies [38,39,43,45] used the Scoliosis Research Society-22 (SRS-22) questionnaire or its revised version (SRS-22r) to assess health-related quality of life; 3 [38,43,45] showed a favorable direction, one showed no clear direction [39], and none showed an unfavorable direction. Chen et al [45] reported that the telerehabilitation group showed improvements in the SRS-22 functional and treatment satisfaction dimensions at 12 months, whereas the corresponding improvements in the control group did not reach statistical significance during the same period; at the 24-month follow-up, both groups showed improvements from baseline across all 5 dimensions (P values ranged from <.001 to .02). Ozger et al [43] reported a statistically significant within-group improvement in the SRS-22 total score in the VR-assisted rehabilitation group (*P*=.01), whereas the control group showed no significant within-group change; the between-group difference was also not significant, and the direction was coded from the asymmetric within-group improvements between the 2 groups. Lau et al [38] reported that the intervention group showed a trend toward greater improvement in the SRS-22r self-image dimension and total score than the control group (interaction *P*=.07; *P*=.09), although neither reached statistical significance; moreover, this study was at high risk of bias, and its results should be interpreted with caution. Tombak et al [39] reported significant within-group improvements in the SRS-22 total score in both groups (both *P*<.001), but the magnitude of improvement was comparable between groups, and the between-group difference was not statistically significant (*P*=.62); this outcome was therefore coded as no clear direction.

The 5-dimensions subgroup analysis (Tables S2–S6 in Multimedia Appendix 5) generally supported the main synthesis direction. The supervision model dimension showed a gradient pattern, with the proportion of studies showing a favorable direction declining as supervision intensity decreased: in the real-time synchronous therapist supervision subgroup, both studies showed a favorable direction [43,45]; in the asynchronous technology-mediated remote monitoring subgroup, one study showed a favorable direction [38]; and in the minimally supervised self-directed practice subgroup, the single study showed no clear direction [39]. This preliminarily suggests that supervision intensity may be one of the moderating factors influencing the direction of effect for quality of life; however, due to the limited number of studies in each subgroup, this is not yet sufficient evidence. Regarding the intervention dose dimension, both studies in the long-term intervention subgroup (total duration >12 weeks) showed a favorable direction [38,45], whereas in the short-term intervention subgroup (≤12 weeks), one of the 2 studies showed a favorable direction [43] and the other showed no clear direction [39]. This suggests that intervention duration may be associated with a favorable direction for quality of life. No systematic directional deviation was observed in the remaining 3 dimensions.

#### Pain

A total of 2 studies reported on pain [37,40], with divergent directions of effect: one showed a favorable direction [37] and the other showed an unfavorable direction [40]. Kisa et al [37] used the Numeric Rating Scale (NRS) to assess joint pain and reported a 3.12-point decrease in the intervention group, which was superior to the 1.56-point decrease in the control group (*P*<.001). Zapata et al [40] used the Numeric Pain Rating Scale (NPRS) to assess low back pain and reported that the in-person supervised group achieved greater pain improvement (a decrease of 3.9 points) than the DVD-only group (a decrease of 2.2 points, *P*=.005); the direction was therefore coded as unfavorable. This unfavorable coding reflects that asynchronous digital educational tools used alone produced a smaller analgesic effect than the in-person supervised control group, rather than indicating that the digital intervention worsened pain. The 2 studies differed fundamentally in pain type (joint pain vs low back pain), intervention content, and comparator intensity, and the directional discrepancy therefore stems largely from this between-study clinical heterogeneity. Given that only 2 studies were available, no subgroup direction-of-effect synthesis was conducted.

#### Body Image

A total of 2 studies [37,39] used the Walter Reed Visual Assessment Scale (WRVAS) to assess body image; one showed a favorable direction [37], one showed no clear direction [39], and none showed an unfavorable direction. Kisa et al [37] reported a 6.04-point decrease in the intervention group, which was superior to the 3.24-point decrease in the control group (*P*=.002; d=1.60). Tombak et al [39] reported statistically significant within-group improvements in both groups, but the magnitude of improvement was comparable between groups, and the between-group difference was not statistically significant (*P*=.68). Given that only 2 studies were available, no subgroup direction-of-effect synthesis was conducted.

### Implementation Outcomes

#### Implementation Outcomes Overview

Implementation outcomes encompass 2 measures: treatment adherence and dropout (loss-to-follow-up rates). Based on a direction-of-effect summary from 11 nonfeasibility studies [17,35-41,43-45] (Table S1 in Multimedia Appendix 5), the proportions of studies showing a favorable direction for the 2 measures were as follows: treatment adherence (n=3, 37.5%) and dropout (loss-to-follow-up rates; n=2, 50.0%). The distribution of effect directions for treatment adherence was relatively dispersed, suggesting that this outcome is markedly moderated by multiple factors; the proportion of studies showing a favorable direction for dropout and loss-to-follow-up rates was slightly higher, but studies showing an unfavorable direction were also present.

#### Treatment Adherence

A total of 8 studies [36-40,43-45] reported on treatment adherence; 3 [43-45] showed a favorable direction, 2 [36,40] showed an unfavorable direction, and 3 [37-39] showed no clear direction, indicating a relatively dispersed distribution of effect directions. Among the studies showing a favorable direction: Chen et al [45] reported that the telerehabilitation group had a higher treatment completion rate (92.6% vs 74.3%) and a higher proportion of participants achieving ≥80% adherence (97.1% vs 60.6%) than the control group (both *P*<.001); Yuan et al [44] reported that the digital intervention group had a higher exercise frequency (5.30 vs 4.63 times/week) and a longer exercise duration (179.44 vs 147.84 min/week) than the control group (both *P*<.001); Ozger et al [43] reported a 100% (n=14) treatment completion rate in the VR-assisted rehabilitation group, with the mean daily step count increasing from 5275 to 8717 (P<.001). Among the studies showing an unfavorable direction: Fishman [36] reported that nonadherence in the remote video group (n=11, 36.7%) was higher than in the in-person outpatient group (n=6 of 30, 20%); however, the reliability of this evidence is limited owing to a serious risk of bias; Zapata et al [40] reported that adherence in the DVD-only group (67%) was lower than in the in-person supervised group (95%, *P*=.001). Among the studies showing no clear direction: Kisa et al [37] used daily monitoring via WhatsApp, and both groups achieved 100% (n=25) treatment completion, yielding a between-group point estimate of zero, so this outcome was coded as no clear direction; in Lau et al [38], a technical failure of the wrist-worn accelerometer produced a low synchronization rate (approximately 15%), so valid adherence monitoring data could not be generated, and this outcome was coded as no clear direction, reflecting a limitation of technical implementation rather than adherence itself; Tombak et al [39] reported nearly identical adherence rates between the 2 groups (97.54% vs 96.89%), and this outcome was coded as no clear direction.

In the 5-dimensions subgroup analysis (Tables S2-S6 in Multimedia Appendix 5), the supervision model dimension showed a gradient pattern, with the proportion of studies showing a favorable direction declining as supervision intensity decreased. In the real-time synchronous therapist supervision subgroup, both studies showed a favorable direction [43,45]; in the asynchronous technology-mediated remote monitoring subgroup, one of the 3 studies showed a favorable direction [44] and 2 showed no clear direction [37,38]; and in the minimally supervised self-directed practice subgroup, none of the 3 studies showed a favorable direction and 2 showed an unfavorable direction [36,40]. This gradient pattern preliminarily suggests that supervision intensity may be one of the moderating factors influencing the direction of effect for treatment adherence. No systematic directional deviation was observed in the remaining 4 dimensions.

#### Dropout and Loss-to-Follow-Up Rates

A total of 4 studies [37,40,44,45] reported dropout and loss-to-follow-up rates; 2 [44,45] showed a favorable direction, one [40] showed an unfavorable direction, and one [37] showed no clear direction. Yuan et al [44] reported that the dropout rate in the digital intervention group (n=7, 10.9%) was lower than that in the control group (n=10, 15.6%). Chen et al [45] reported that the dropout rate in the telerehabilitation group (n=2, 5.9%) was lower than that in the self-practice group (n=5, 15.2%); the point estimate indicated a favorable trend, but the between-group difference was not statistically significant (*P*=.26). Zapata et al [40] reported that the dropout rate in the DVD-only group (n=9, 35%) was higher than that in the face-to-face supervision group (n=2, 11%), with the direction coded as unfavorable. Kisa et al [37] found zero dropouts in both groups (0% vs 0%), with a point estimate difference of zero, coded as no clear direction.

In the 5-dimensions subgroup analysis (Tables S2-S6 in Multimedia Appendix 5), the directional distribution patterns for the supervision mode dimension were consistent with the adherence outcomes: in the asynchronous technology-mediated remote monitoring subgroup, one study [44] showed a favorable direction and one [37] showed no clear direction; one study [40] in the minimal supervision self-practice subgroup showed an unfavorable trend, suggesting a potential association between supervision intensity and dropout (loss-to-follow-up rates). No systematic directional patterns were identified for the remaining dimensions.

### Sensitivity Analysis

A prespecified sensitivity analysis restricted the direction-of-effect synthesis to the 9 studies not rated as high risk of bias (RoB 2) or serious risk of bias (ROBINS-I), excluding Fishman [36] and Lau et al [38], to assess the extent to which the main synthesis conclusions relied on studies of lower methodological quality (Table S7 in Multimedia Appendix 5). The favorable-direction signal for the Cobb angle was strengthened after exclusion (71.4% → 80.0%), and the study with an unfavorable direction (Andrade et al [17]) was retained, suggesting that this signal does not depend on studies at high or serious risk of bias and is therefore robust. The proportion of studies showing a favorable direction for health-related quality of life decreased (75.0% → 66.7%), but the 2 retained studies [43,45] still showed a favorable direction, and the signal persisted overall. The proportion of studies showing a favorable direction for treatment adherence increased (37.5% → 50.0%), and the unfavorable signal from Zapata et al [40] persisted, confirming robustness. For the remaining 7 outcomes, the direction-of-effect proportions were unchanged in the sensitivity analysis.

### Certainty of Evidence

Using the GRADE approach, we assessed the certainty of evidence for each of the 10 outcomes included in the direction-of-effect synthesis (Table S8 in Multimedia Appendix 5): only the Cobb angle had moderate certainty; spinal flexibility, respiratory function, postural control and gait, and health-related quality of life had low certainty; and ATR, pain, body image, treatment adherence, and dropout and loss-to-follow-up rates had very low certainty. The certainty of the available evidence was generally low, with downgrades primarily attributed to imprecision and risk of bias, followed by inconsistency in effect directions; indirectness affected only a few outcomes.

### Qualitative Description of Feasibility Study Outcomes

Wan et al [34] developed an optical sensor-based (Vicon system) motion-sensing video game system for ADS (n=10), in which patients controlled game characters by adjusting their trunk posture in real time. After a 6-week intervention (3 sessions/week), immediate improvements in participants’ sagittal plane posture were recorded following each training session. After 6 weeks, improvements were observed in shoulder and pelvic tilt angles, and postural awareness was enhanced; however, no significant improvement in muscle symmetry was observed. Nam et al [42] developed an augmented reality smart-bar device integrated with a 9-axis sensor for AIS (n=13), which, in conjunction with a mobile app, provided real-time AR posture framework overlays and audio error feedback. Compared with the no-AR condition, AR guidance reduced movement deviation angles, postural sway was suppressed to some extent, and movement accuracy was improved.

### Safety and Adverse Reactions

None of the 13 included studies reported any serious adverse events. Mild discomfort was reported in 2 studies. In Moraes et al [41], 2 participants experienced transient dizziness after prolonged VR headset use, which resolved spontaneously after rest; in Wan et al [34], one participant experienced mild muscle soreness after training, which resolved after rest or adjustment of exercise intensity.

## Discussion

### Principal Findings

To our knowledge, this systematic review represents the first structured synthesis of evidence on the application of digital health technologies in scoliosis rehabilitation using a technology classification framework. A total of 13 studies (574 patients) were included, and the review followed the SWiM reporting guideline [24], synthesizing evidence across 4 outcome domains using a vote-counting method based on the direction of effect [31,32].

In the clinical outcomes domain, digital interventions generally showed a favorable direction for the Cobb angle and spinal flexibility, whereas the evidence for ATR correction was weaker, with most studies showing no clear direction. This pattern suggests that improvements in overall spinal alignment and flexibility are more reliable, whereas the effect of segmental rotational deformities remains to be confirmed. In the functional outcomes domain, the favorable direction was highly consistent for respiratory function and postural control, reflecting the potential value of digital interventions in the functional dimension. Regarding patient-reported outcomes, health-related quality of life showed a relatively consistent favorable direction, whereas the evidence for pain and body image was inconsistent, indicating that the impact of digital interventions on subjective experiences remains variable. In the implementation outcomes domain, the effect directions were the most dispersed; the proportion of studies showing a favorable direction for treatment adherence was relatively low, and dropout and loss-to-follow-up rates likewise showed no consistent advantage. In summary, the potential benefits of digital interventions for spinal morphology and function are relatively clear, whereas their sustained and standardized use (particularly as represented by adherence) may be a key factor influencing whether clinical benefits can be realized. It should be noted that the included studies exhibit substantial clinical heterogeneity, and the GRADE assessment indicated low certainty of the overall evidence. Therefore, the synthesized findings for each outcome should be interpreted cautiously as preliminary and exploratory evidence.

Subgroup analyses revealed several interpretable patterns. First, the favorable direction for the Cobb angle was most evident when digital interventions were compared with unsupervised home exercise, whereas this advantage diminished when they were compared with in-person supervised rehabilitation. This suggests that their added value lies primarily in serving as a substitute for or supplement to unsupervised training, rather than surpassing standardized in-person rehabilitation. Second, improvements in ATR were more commonly observed in studies with longer intervention durations (>12 weeks), suggesting that an adequate intervention period may be a critical factor in correcting rotational deformities. Third, the favorable direction for health-related quality of life and treatment adherence diminished as supervision intensity decreased, suggesting that the intensity of professional supervision may be a common factor influencing both long-term adherence and subjective benefits. Sensitivity analyses further indicated that these key findings remained generally robust after excluding studies at high or serious risk of bias. Taken together, the directional evidence and subgroup patterns indicate that the principal barrier to the clinical translation of digital interventions may lie less in efficacy itself than in patients’ ability to sustain use, with adherence being a key manifestation. This is consistent with a perspective increasingly emphasized in the digital rehabilitation literature: the real-world effectiveness of a technology depends largely on its implementation pathway and on sustained user engagement [47,48].

### Technical Tiering of Digital Interventions

#### Overview

The 3-tier classification framework was developed through systematic analysis of the included evidence base, with tier assignment guided by dimensions established in the National Institute for Health and Care Excellence (NICE) Evidence Standards Framework for Digital Health Technologies [49] and the World Health Organization (WHO) Classification of Digital Interventions, Services and Applications in Health [50]. The mature application tier comprises technologies supported by multiple independent RCTs with consistent clinical outcomes, implemented through consumer-grade platforms requiring no specialized hardware, and applicable within established protocols for routine clinical or home-based rehabilitation. The emerging technology tier encompasses technologies with a controlled but heterogeneous evidence base, reliant on commercially available specialized hardware, and predominantly deployed within research or specialist clinical environments. The frontier exploration tier designates technologies characterized by a nascent independent evidence base—whether reflecting limited study volume, primary deployment as secondary rather than standalone intervention components, or custom-built platform architectures that preclude standardized replication across clinical settings—for which no established clinical protocols currently exist. These criteria are intended to make tier assignment transparent and replicable across different evidence contexts.

#### Mature Application Tier

Synchronous remote rehabilitation via conventional communication software (WhatsApp and Zoom), DVD, and video instruction [17,35-37,39,40,45] represent widely adopted digital interventions in scoliosis treatment. These technologies require no specialized medical equipment; patients need only a smartphone or tablet to participate in training, maintaining low technical barriers and minimal financial burden [51]. During the COVID-19 pandemic, remote rehabilitation models gained global adoption, demonstrating their feasibility in maintaining rehabilitation continuity [17,52,53]. For patients in medically underserved remote areas, this technology also provides accessible professional rehabilitation guidance [54,55]. However, accessibility does not guarantee clinical efficacy. Key limitations include lack of guidance and supervision with asynchronous video instruction; potential failure to adequately capture compensatory postures during corrective training in synchronous video sessions; and low patient adherence, making long-term maintenance challenging [17,37]. Based on these limitations, we suggest that this technology is better suited as a complement to, rather than a substitute for, more advanced digital interventions or traditional in-person training and is more effectively applied for scoliosis education and posture management reminders. This aligns with findings from Molina-Garcia et al [56], whose review of 37 clinical trials on telerehabilitation revealed that although telerehabilitation outperformed conventional controls, its advantages were no longer significant when directly compared to in-person rehabilitation. This suggests that telerehabilitation should serve as an alternative when in-person rehabilitation is inaccessible, rather than as the primary intervention model.

#### Emerging Technology Tier

VR and AR technologies integrate computer-generated 3D virtual environments with real-world settings in real time to provide patients with biofeedback and interactive training experiences, with core advantages in multisensory channel integration, instantaneous movement correction, and gamified task design [57,58]. This technology has shown preliminary therapeutic benefit in scoliosis treatment, including improved spinal flexibility [43], reduced movement deviations during corrective training [42], and prolonged standing endurance with improved seated stability [41]. A core challenge in PSSE is rotational breathing training (rotational angular breathing), where patients must master an asymmetric breathing pattern of “inhale and expand on the concave side, exhale and contract on the convex side” [59]. However, without supervision, accurately sensing this pattern is difficult, leading to movement distortion and suboptimal training outcomes [60,61]. We hypothesize that VR and AR systems could visualize bilateral respiratory amplitude in real time through a virtual 3D lung model, converting proprioceptive feedback into intuitive visual cues [62], which may help patients identify deviations and actively correct them, thereby potentially lowering the learning threshold for 3D corrective movements. Recent reviews further suggest that this technology may promote motor learning and functional recovery, offering unique advantages for mastering complex movement patterns [63]. Wearable sensor technologies enable continuous monitoring of kinematic parameters and real-time alerts for postural deviations, providing objective assessment tools for home-based rehabilitation [64-66]. However, the evidence for this technology remains limited. For instance, Lau et al [38] reported failure of adherence monitoring due to low synchronization rates with accelerometers. Currently, these technologies remain in exploratory pilot phases, with high costs and maintenance expenses limiting their widespread adoption [67].

#### Frontier Exploration Tier

Yuan et al [44] developed a comprehensive digital health management platform for scoliosis, integrating AI-based automatic classification, personalized treatment matching, 3-tiered remote supervision, and data feedback. This platform unifies assessment, treatment, monitoring, and feedback, representing a promising future direction for digital interventions. Results from a 6-month intervention demonstrated not only improved Cobb angles but also increased patient-initiated exercise frequency and correction adherence rates. This suggests that enhancing scoliosis treatment outcomes relies not only on technological advancements but critically on activating patients’ intrinsic motivation for training. This perspective is supported by a large-scale umbrella review [68]. The effectiveness of digital health interventions is primarily driven by behavioral change techniques, where feedback monitoring, goal setting, personalization, and human guidance are key elements for enhancing adherence and activating intrinsic motivation.

### Clinical Value and Significance of Digital Interventions in Scoliosis

It should be noted that the mechanisms discussed in this section are interpretations based on existing theories rather than conclusions directly confirmed by research; since most included studies did not measure neurophysiological or behavioral mediators, the following discussion should be understood as a theoretical explanation rather than an empirical finding.

#### Real-Time Feedback and Motor Learning

Patients with scoliosis often exhibit proprioceptive abnormalities due to long-term asymmetric loading, specifically manifested as deviations in spinal spatial orientation and compensatory movement patterns [69-71]. Based on this, it is hypothesized that real-time, precise biomechanical feedback can facilitate patients’ recognition and active correction of incorrect movement patterns, thereby compensating for proprioceptive deficits and aiding in the establishment of correct movement patterns [72-74]. Wan et al [34] used a gamified visual feedback system for monitoring: when the patient’s posture deviated, the system automatically slowed the game’s pace to prompt active adjustment; this external feedback may have facilitated motor learning processes related to error detection, thereby promoting immediate postural correction. In that study, after 6 weeks of training, patients showed improvements not only in sagittal balance but also in coronal-plane parameters not directly targeted by the game, suggesting that overall postural awareness may be important for patients with scoliosis. Nam et al [42] further used an augmented reality-overlaid postural framework to display real-time deviation angles for spinal lateral flexion or rotation, supplemented by audio cues, and suggested that visual feedback may aid proprioceptive integration. Compared with traditional mirror-based training, real-time dynamic feedback theoretically offers 3 advantages: first, 3D visualization eliminates the blind spots inherent in 2D mirror images; second, quantified deviation-angle data are more precise than subjective perception; and third, multimodal (visual and auditory) feedback may reduce cognitive load and support motor learning, thereby enhancing patients’ ability to correct abnormal postures [75,76]. Notably, existing studies have primarily focused on immediate training effects [34,42], while questions regarding whether movement patterns can be maintained after feedback is withdrawn, as well as the optimal frequency and intensity of feedback, remain to be investigated. In fact, more frequent feedback does not necessarily facilitate motor learning: research outside the field of scoliosis indicates that intermittent rather than continuous feedback is more conducive to the long-term retention of movement patterns [77]. Therefore, clarifying how often and how intensively feedback should be delivered may have implications for the long-term efficacy of digital interventions.

#### Improving Patient Adherence

Patient adherence is a key factor influencing the effectiveness of rehabilitation [78]. As with the mechanisms discussed above, the following account is a theory-based interpretation rather than a directly tested finding. Self-Determination Theory (SDT) posits that autonomy, competence, and relatedness are the 3 fundamental psychological needs that drive human behavior [79,80]; a meta-analysis of SDT-informed interventions in the health domain found that supporting these 3 psychological needs improves motivation and health behavior change [81]; digital interventions that target these needs may therefore enhance adherence. Digital interventions that integrate gamification with remote monitoring may stimulate intrinsic motivation and enhance rehabilitation adherence. The adaptive difficulty algorithm developed by Nam et al [42] may address the “competence” need by avoiding the boredom caused by excessive simplicity and the frustration caused by excessive difficulty; reward mechanisms such as coin collection and battery charging may activate immediate gratification and thereby promote sustained training behavior. However, the motivational benefits of gamification may not be sustained. Moraes et al [41] observed a decline in certain metrics by the fourth training session, suggesting that repetitive stimuli may attenuate motivation. Therefore, periodic updates to game scenarios and tasks, along with the establishment of progressive long-term goals, may help maintain a sense of autonomy. Lau et al [38] used a HIIT video library that randomly generated sequences from 40 exercises, which theoretically could prevent monotony; however, the low accelerometer synchronization rate led to failed adherence monitoring, highlighting the challenges of implementing motivational design in practice.

The concept of “self-efficacy” proposed by social cognitive theory provides a theoretical basis for the benefits of digital interventions [82-84]. A systematic review and meta-analysis by Newby et al [85] reported that digital health interventions can significantly enhance self-efficacy. When patients observe objective progress data (such as increased step counts or reduced Cobb angles) through digital platforms, this visual feedback may reinforce their self-efficacy, creating a positive cycle of “success experiences → increased confidence → sustained behavior” [42,44,86]. A systematic review and meta-analysis [87] reported that feedback monitoring and self-monitoring were among the most frequently used and effective techniques in digital behavior change interventions (applied in 94.4% and 88.9% of studies, respectively) and were important for sustaining behavioral change. By contrast, interventions that provide limited or delayed feedback, irrespective of whether they are delivered digitally or conventionally, may make it harder for patients to perceive treatment efficacy and may increase the likelihood of discontinuation; the higher dropout rate in the unsupervised DVD-only group (35%, n=9) than in the supervised group (11%, n=2) reported by Zapata et al [40] is consistent with this.

### Implementation Across Digital Technologies: the Role of Supervision

A key finding of this review is that, in digital scoliosis rehabilitation, whether an intervention can be sustained and produce clinical benefits in the real world may depend primarily on the level of human supervision embedded in its delivery, rather than on the technology category itself. A cross-comparison of implementation outcomes across the 5 technology categories revealed that the proportion of studies showing a favorable direction for treatment adherence and dropout (loss-to-follow-up rates) did not increase with the level of technological sophistication (Table S2 in Multimedia Appendix 5). Notably, even within the same technology category (synchronous telerehabilitation), implementation outcomes varied depending on the supervision model. Together, these observations suggest that implementation success depends less on the technology category than on the degree of professional supervision. This is consistent with recent digital-health evidence that adherence is multifactorial, with support from health care professionals identified as a key determinant [88]. The role of supervision can be understood at 2 levels. First, supervision directly influences treatment efficacy by ensuring the quality of exercise execution [40,89]. Second, supervision sustains long-term patient engagement by providing accountability and feedback—a function particularly important in the conservative management of scoliosis, which relies on sustained training [90,91]. Importantly, this reliance is not absolute: one study reported that a home-based program relying primarily on monthly supervision still achieved outcomes comparable to clinic-supervised training for most measures [39].

Overall, supervision intensity, as a key determinant of implementation effectiveness [92,93], serves as an important complement to the technology classification framework proposed in this review. While the technology classification defines the functional capabilities an intervention can provide, whether these capabilities translate into sustained clinical benefits may depend substantially on the corresponding supervision framework. Previous evidence indicates that adequate supervision in the early stages helps foster long-term home adherence [94] and that remote supervision under professional guidance can achieve outcomes comparable to in-person rehabilitation [56]. Accordingly, this review suggests that a “hybrid model combined with phased supervision intensity” may represent a promising strategy for balancing efficacy, adherence, and generalizability, namely, transitioning from early intensive in-person guidance [35] to high-frequency, digitally supported remote supervision during the maintenance phase [44], and sustaining efficacy through low-frequency reassessment during long-term follow-up [39]. However, due to the limited number of included studies across different technology categories and subgroups, as well as the lack of head-to-head comparisons, the aforementioned strategy remains an exploratory hypothesis at this stage. The optimal supervision intensity and timing of transition require validation through prospective studies.

### Limitations and Future Development

This review has several limitations. (1) The available evidence is limited in both quantity and quality: research in this field remains scarce, with only 13 studies included; among the 7 RCTs, only 42.9% (n=3) were at low risk of bias, while 33.3% (n=2) of the 6 nonrandomized studies had a serious risk of bias, and sample sizes were generally small; moreover, reporting on intervention fidelity and adherence monitoring was insufficient, and acceptability and feasibility were formally assessed with validated instruments in only one study [17], precluding a quantitative comparison of these implementation outcomes across technologies. (2) Follow-up was predominantly short- to medium-term, with only one study reaching 24 months [45]; given the progressive nature of scoliosis and the need for sustained conservative management until skeletal maturity, whether the observed improvements persist beyond the active treatment period remains uncertain. (3) As no meta-analysis was performed, the review provides no pooled effect estimates, so the magnitude and precision of effects could not be quantified, and statistical tests for publication bias (eg, funnel plots and Egger test) were not applicable; reporting and publication biases therefore cannot be excluded. Correspondingly, the GRADE-rated certainty of the evidence was generally low. (4) Generalizability is constrained: the included studies originated predominantly from middle- and high-income countries, with a lack of data from low-income nations, and the restriction to English-language publications, with 4 of the 13 studies originating from Chinese institutions, may have systematically excluded non-English, particularly East Asian, literature, thereby underrepresenting the full breadth of current evidence.

Future research should conduct large-sample, long-term follow-up multicenter RCTs to validate the long-term efficacy and safety of digital interventions for scoliosis. Additionally, optimal implementation strategies based on patient characteristics should be explored to achieve personalized, precise interventions. Further health economic evaluations are needed to compare the cost-effectiveness of different digital technologies versus traditional rehabilitation. Future reviews should also incorporate multilingual search strategies to achieve more geographically and linguistically representative evidence synthesis. As new technologies emerge, digital interventions in scoliosis management should evolve alongside them, continuously integrating these advances to further enhance efficacy and patient experience.

### Conclusion

This review systematically evaluated the application of digital health technologies in scoliosis rehabilitation: across the 13 included studies, a vote-counting synthesis based on the direction of effect indicated a favorable direction for digital interventions in spinal alignment and flexibility, respiratory function, postural control and gait, and health-related quality of life; however, the certainty of the evidence was low. Its innovation lies in being the first to incorporate diverse digital interventions into a technology classification framework comprising 5 technology categories and 3 maturity tiers, integrating cross-technology evidence of effectiveness. Based on this, the review suggests that whether these benefits can be realized in the real world may depend not only on the technology itself but also on the supervision models that sustain patient adherence. It is precisely this cross-technology integration that distinguishes this review from previous similar reviews, which often focused on a single technology or a single function. Consequently, this review not only provides a structured and actionable basis for clinical technology selection but also shifts the focus from “which technology is most effective” to “how to ensure sustained use of the technology in practice.” At the clinical level, this implies that technologies may be selected according to their maturity tier and paired with appropriate supervision strategies (such as a “hybrid model combined with phased supervision intensity”) to ensure adherence. This approach could expand access to high-quality rehabilitation resources in underdeveloped and remote areas without compromising therapeutic efficacy. Adequately powered studies with sufficient follow-up are still needed to confirm the value of digital interventions in precision rehabilitation for scoliosis.

## Supplementary material

10.2196/91461 Multimedia Appendix 1 Literature search strategies and results for all databases.

10.2196/91461 Multimedia Appendix 2 List of studies excluded at the full-text assessment stage, with reasons for exclusion.

10.2196/91461 Multimedia Appendix 3 Assessment of intervention reporting completeness for the included studies using the TIDieR (Template for Intervention Description and Replication) checklist.

10.2196/91461 Multimedia Appendix 4 Extracted outcome data for the included nonfeasibility studies.

10.2196/91461 Multimedia Appendix 5 Direction-of-effect synthesis tables, subgroup analyses, sensitivity analysis, and Grading of Recommendations Assessment, Development, and Evaluation (GRADE) certainty-of-evidence assessment.

10.2196/91461 Checklist 1 PRISMA 2020 expanded.

10.2196/91461 Checklist 2 PRISMA-S checklist. 91461-Multimedia-Appendix-7.pdf

10.2196/91461 Checklist 3 SWiM checklist. 91461-Multimedia-Appendix-8.pdf

10.2196/91461 Checklist 4 PRISMA 2020 for Abstracts checklist.

## Abbreviations

ADS: adult degenerative scoliosis
AIS: adolescent idiopathic scoliosis
AR: augmented reality
ATR: angle of trunk rotation
BCT: behavior change technique
CAD: computer-aided design
CAM: computer-aided manufacturing
DVD: digital versatile disc
FEV1%: forced expiratory volume in 1 second, percent predicted
FVC%: forced vital capacity, percent predicted
GRADE: Grading of Recommendations Assessment, Development and Evaluation
HIIT: high-intensity interval training
JIA: juvenile idiopathic arthritis
JIS: juvenile idiopathic scoliosis
MCID: minimal clinically important difference
MEP: maximal expiratory pressure
MIP: maximal inspiratory pressure
NICE: National Institute for Health and Care Excellence
NPRS: Numeric Pain Rating Scale
NRS: Numeric Rating Scale
PEF%: peak expiratory flow, percent predicted
PICOS: Population, Intervention, Comparator, Outcome, Study Design
PRESS: Peer Review of Electronic Search Strategies
PRISMA: Preferred Reporting Items for Systematic Reviews and Meta-Analyses
PRISMA-S: Preferred Reporting Items for Systematic Reviews and Meta-Analyses literature search extension
PROSPERO: International Prospective Register of Systematic Reviews
PSF: posterior spinal fusion
PSSE: physiotherapeutic scoliosis-specific exercises
PUMCH-SSE: Peking Union Medical College Hospital Scoliosis-Specific Exercise
RCT: randomized controlled trial
RoB 2: Risk of Bias Assessment Tool Version 2
ROBINS-I: Risk of Bias in Nonrandomized Studies of Interventions
SDT: Self-Determination Theory
SRS-22: Scoliosis Research Society-22
SRS-22r: Scoliosis Research Society-22 revision
SVA: sagittal vertical axis
SWiM: Synthesis Without Meta-analysis
TIDieR: Template for Intervention Description and Replication
TSK: Tampa Scale for Kinesiophobia
VR: virtual reality
WHO: World Health Organization
WRVAS: Walter Reed Visual Assessment Scale
