# Supplementary material for: Application of Digital Health Technologies in Scoliosis Rehabilitation: Systematic Review Based on the Technology Classification Framework
Source: J Med Internet Res. 2026 Aug 28;28:e91461. doi: 10.2196/91461 (PMC13524367; doi:10.2196/91461)
Supplement: Supplementary file 3 [file jmir-v28-e91461-s001.docx]

**Literature Search Strategies and Results**

1. **Pubmed (Medline): 1211 studies**

**Keywords:**

**Digital Health**

**Telemedicine**

**Mobile Applications**

**InternetBased Intervention***

**Information Technolog***

**Digital Technolog***

**Scoliosis***

**exercise**

**rehabilitation**

**PSSE**

**search strategy:**

((((((((digital health[MeSH Terms]) OR (Telemedicine[MeSH Terms])) OR (Mobile Applications[MeSH Terms])) OR (Internet Based Intervention*[MeSH Terms])) OR (Information Technolog*[MeSH Terms])) OR (Digital Technolog*[MeSH Terms])) AND (Scoliosis*[MeSH Terms])) AND (exercise* OR rehabilitation OR PSSE)) OR ((("Digital Health"[Title/Abstract] OR "Telemedicine"[Title/Abstract] OR "Mobile Applications"[Title/Abstract] OR "Internet Based Intervention*"[Title/Abstract] OR "Information Technolog*"[Title/Abstract] OR "Digital Technolog*"[Title/Abstract] OR "Mobile Health"[Title/Abstract] OR "Tele-Care"[Title/Abstract] OR "Tele-ICU"[Title/Abstract] OR "Tele-Intensive Care"[Title/Abstract] OR "Tele-Referral"[Title/Abstract] OR Telecare[Title/Abstract] OR Telehealth[Title/Abstract] OR "Virtual Medicine*"[Title/Abstract] OR eHealth[Title/Abstract] OR mHealth[Title/Abstract] OR Telecommunication*[Title/Abstract] OR Teleconference*[Title/Abstract] OR Telegraph*[Title/Abstract] OR "distance counsel*"[Title/Abstract] OR remote[Title/Abstract] OR telerehabilitation[Title/Abstract] OR telephone[Title/Abstract] OR "cell phone"[Title/Abstract] OR videoconferencing[Title/Abstract] OR "wireless technology"[Title/Abstract] OR "Mobile Apps"[Title/Abstract] OR "Portable Electronic"[Title/Abstract] OR "Portable Software"[Title/Abstract] OR Smartphone[Title/Abstract] OR Computer*[Title/Abstract] OR Mobile*[Title/Abstract] OR "Internet based"[Title/Abstract] OR internet*[Title/Abstract] OR web[Title/Abstract] OR online[Title/Abstract] OR Digital Intervention*[Title/Abstract] OR Digital[Title/Abstract] OR Wearable[Title/Abstract] OR Exergame*[Title/Abstract] OR Game*[Title/Abstract]) AND (Scoliosis*[Title/Abstract] OR "Idiopathic Scoliosis"[Title/Abstract] OR "Adolescent Idiopathic Scoliosis"[Title/Abstract] OR "Juvenile Idiopathic Scoliosis"[Title/Abstract] OR "Spinal Curvature*"[Title/Abstract] OR Kyphosis*[Title/Abstract] OR Hyperkyphosis*[Title/Abstract] OR Lordosis*[Title/Abstract])) AND (exercise*[Title/Abstract] OR Physica*[Title/Abstract] OR Aerobic*[Title/Abstract] OR Isometric*[Title/Abstract] OR Activit*[Title/Abstract] OR rehabilitation*[Title/Abstract] OR train*[Title/Abstract] OR sport*[Title/Abstract] OR intervention*[Title/Abstract] OR treatment*[Title/Abstract] OR therapy*[Title/Abstract] OR "Schroth"[Title/Abstract] OR "SEAS"[Title/Abstract] OR "DoboMed"[Title/Abstract] OR "Side Shift"[Title/Abstract] OR "Lyon"[Title/Abstract] OR "BSPTS"[Title/Abstract] OR "FITS"[Title/Abstract] OR "Stabilization"[Title/Abstract]))

2. **IEEE Xplore：426 studies**

**search strategy:**

("All Metadata":"Digital Health" OR "All Metadata":"Telemedicine" OR "All Metadata":"Mobile Applications" OR "All Metadata":"Internet Based Intervention" OR "All Metadata":"Information Technology" OR "All Metadata":"Digital Technology" OR "All Metadata":"Mobile Health" OR "All Metadata":"Tele-Care" OR "All Metadata":"Tele-ICU" OR "All Metadata":"Tele-Intensive Care" OR "All Metadata":"Tele-Referral" OR "All Metadata":Telecare OR "All Metadata":Telehealth OR "All Metadata":"Virtual Medicine" OR "All Metadata":eHealth OR "All Metadata":mHealth OR "All Metadata":Telecommunication OR "All Metadata":Teleconference OR "All Metadata":Telegraph* OR "All Metadata":"distance counsel*" OR "All Metadata":remot* OR "All Metadata":telerehabilitation OR "All Metadata":telephone OR "All Metadata":"cell phone" OR "All Metadata":videoconferencing OR "All Metadata":"wireless technology" OR "All Metadata":"Mobile Apps" OR "All Metadata":"Portable Electronic" OR "All Metadata":"Portable Software" OR "All Metadata":Smartphone OR "All Metadata":Computer* OR "All Metadata":Mobile OR "All Metadata":"Internet based" OR "All Metadata":internet* OR "All Metadata":web OR "All Metadata":online OR "All Metadata":Digital Intervention OR "All Metadata":Digital* OR "All Metadata":Wearable OR "All Metadata":Exergame* OR "All Metadata":Game*) AND ("All Metadata":Scoliosis* OR "All Metadata":"Idiopathic Scoliosis" OR "All Metadata":"Adolescent Idiopathic Scoliosis" OR "All Metadata":"Juvenile Idiopathic Scoliosis" OR "All Metadata":"Spinal Curvature*" OR "All Metadata":Kyphosis* OR "All Metadata":Hyperkyphosis* OR "All Metadata":Lordosis*) AND ("All Metadata":exercise* OR "All Metadata":Physica* OR "All Metadata":Aerobic* OR "All Metadata":Isometric* OR "All Metadata":Activit* OR "All Metadata":rehabilitation OR "All Metadata":train* OR "All Metadata":sport* OR "All Metadata":intervention* OR "All Metadata":treatment* OR "All Metadata":therapy* OR "All Metadata":"Schroth" OR "All Metadata":"SEAS" OR "All Metadata":"DoboMed" OR "All Metadata":"Side Shift" OR "All Metadata":"Lyon" OR "All Metadata":"BSPTS" OR "All Metadata":"FITS" OR "All Metadata":"Stabilization")

3. **Embase：1973 studies**

**search strategy:**

('telemedicine':ti,ab,kw OR 'mobile applications':ti,ab,kw OR 'internet based intervention':ti,ab,kw OR 'information technology':ti,ab,kw OR 'digital technology':ti,ab,kw OR 'mobile health':ti,ab,kw OR 'tele-care':ti,ab,kw OR 'tele-icu':ti,ab,kw OR 'tele-intensive care':ti,ab,kw OR 'tele-referral':ti,ab,kw OR telecare:ti,ab,kw OR telehealth:ti,ab,kw OR 'virtual medicine':ti,ab,kw OR ehealth:ti,ab,kw OR mhealth:ti,ab,kw OR telecommunication:ti,ab,kw OR teleconference:ti,ab,kw OR telegraph*:ti,ab,kw OR 'distance counsel*':ti,ab,kw OR remot*:ti,ab,kw OR telerehabilitation:ti,ab,kw OR telephone:ti,ab,kw OR 'cell phone':ti,ab,kw OR videoconferencing:ti,ab,kw OR 'wireless technology':ti,ab,kw OR 'mobile apps':ti,ab,kw OR 'portable electronic':ti,ab,kw OR 'portable software':ti,ab,kw OR smartphone:ti,ab,kw OR computer*:ti,ab,kw OR mobile:ti,ab,kw OR 'internet based':ti,ab,kw OR internet*:ti,ab,kw OR web:ti,ab,kw OR online:ti,ab,kw OR 'digital intervention':ti,ab,kw OR digital*:ti,ab,kw) AND (scoliosis*:ti,ab,kw OR 'idiopathic scoliosis':ti,ab,kw OR 'adolescent idiopathic scoliosis':ti,ab,kw OR 'juvenile idiopathic scoliosis':ti,ab,kw OR 'spinal curvature*':ti,ab,kw OR kyphosis*:ti,ab,kw OR hyperkyphosis*:ti,ab,kw OR lordosis*:ti,ab,kw) AND (exercise*:ti,ab,kw OR physica*:ti,ab,kw OR aerobic*:ti,ab,kw OR isometric*:ti,ab,kw OR activit*:ti,ab,kw OR rehabilitation:ti,ab,kw OR train*:ti,ab,kw OR sport*:ti,ab,kw OR intervention*:ti,ab,kw OR treatment*:ti,ab,kw OR therapy*:ti,ab,kw OR 'schroth':ti,ab,kw OR 'seas':ti,ab,kw OR 'dobomed':ti,ab,kw OR 'side shift':ti,ab,kw OR 'lyon':ti,ab,kw OR 'bspts':ti,ab,kw OR 'fits':ti,ab,kw OR 'stabilization':ti,ab,kw)

4. **Web of Science：1952 studies**

**search strategy:**

"Digital Health" OR "Telemedicine" OR "Mobile Applications" OR "Internet Based Intervention*" OR "Information Technolog*" OR "Digital Technolog*" OR "Mobile Health" OR "Tele-Care" OR "Tele-ICU" OR "Tele-Intensive Care" OR "Tele-Referral" OR Telecare OR Telehealth OR "Virtual Medicine*" OR eHealth OR mHealth OR Telecommunication* OR Teleconference* OR Telegraph* OR "distance counsel*" OR remote OR telerehabilitation OR telephone OR "cell phone" OR videoconferencing OR "wireless technology" OR "Mobile Apps" OR "Portable Electronic" OR "Portable Software" OR Smartphone OR Computer* OR Mobile* OR "Internet based" OR internet* OR web OR online OR Digital Intervention* OR Digital OR Wearable OR Exergame* OR Game* (All Fields) and Scoliosis* OR "Idiopathic Scoliosis" OR "Adolescent Idiopathic Scoliosis" OR "Juvenile Idiopathic Scoliosis" OR "Spinal Curvature*" OR Kyphosis* OR Hyperkyphosis* OR Lordosis* OR "Spinal Deformity" (All Fields) and exercise* OR Physica* OR Aerobic* OR Isometric* OR Activit* OR rehabilitation* OR train* OR sport* OR intervention* OR treatment* OR therapy* OR "Schroth" OR "SEAS" OR "Side Shift" OR "Lyon" OR "BSPTS" OR "FITS" OR "Stabilization" (All Fields)
