# Supplementary material for: Application of Digital Health Technologies in Scoliosis Rehabilitation: Systematic Review Based on the Technology Classification Framework
Source: J Med Internet Res. 2026 Aug 28;28:e91461. doi: 10.2196/91461 (PMC13524367; doi:10.2196/91461)
Supplement: Supplementary file 4 [file jmir-v28-e91461-s002.docx]

# List of Excluded Studies at Full-Text Screening Stage

# This supplementary table provides a comprehensive list of studies excluded at the full-text assessment stage, categorized by exclusion reason to enhance transparency and reproducibility of the systematic review process.

| **No.** | **Author, Year** | **Title** | **Reason*** | **Specific Details** |
| --- | --- | --- | --- | --- |
| 1 | Li et al., 2024 | Prevalence of scoliosis in children and adolescents: a systematic review and meta-analysis | R1 | Systematic review and meta-analysis |
| 2 | Wang et al., 2025 | Global prevalence and associated risk factors of scoliosis in children and adolescents: a systematic review | R1 | Systematic review |
| 3 | Romano et al., 2024 | Therapeutic exercises for idiopathic scoliosis in adolescents | R1 | Cochrane systematic review |
| 4 | Kuru Çolak et al., 2025 | Systematic Review of Clinical Outcome Parameters of Conservative Treatment of Adolescent Idiopathic Scoliosis Patients | R1 | Systematic review focused on outcome measures |
| 5 | Li et al., 2025 | Behavior Change Strategies in Digital Exercise Interventions for Adolescent Idiopathic Scoliosis: Scoping Review | R1 | Scoping review |
| 6 | Zhou et al., 2021 | The effects of exercise therapy on adolescent idiopathic scoliosis: an overview of systematic reviews | R1 | Overview of systematic reviews |
| 7 | Negrini et al., 2018 | 2016 SOSORT guidelines: orthopaedic and rehabilitation treatment of idiopathic scoliosis during growth | R1 | Clinical guideline |
| 8 | Lightsey et al., 2021 | The past, present, and future of remote patient monitoring in spine care: an overview | R1 | Narrative overview |
| 9 | Park et al., 2021 | Effectiveness of interactive augmented reality-based telerehabilitation: protocol | R1 | Study protocol only |
| 10 | Berdishevsky et al., 2016 | Physiotherapy scoliosis-specific exercises—a comprehensive review | R1 | Comprehensive review |
| 11 | Ren et al., 2025 | Comparative efficacy of conservative interventions for adolescent idiopathic scoliosis: network meta-analysis | R1 | Network meta-analysis |
| 12 | de Sèze & Cugy, 2012 | Pathogenesis of idiopathic scoliosis: a review | R1 | Narrative review |
| 13 | Singh et al., 2025 | Lifestyle eHealth and mHealth Interventions for Children and Adolescents: Systematic Umbrella Review | R1 | Umbrella review |
| 14 | Baroni et al., 2023 | The state of the art in telerehabilitation for musculoskeletal conditions | R1 | Narrative review |
| 15 | Kalron et al., 2020 | The Design, Development, and Testing of a Virtual Reality Device for Upper Limb Training in People With Multiple Sclerosis | R2 | Multiple sclerosis patients, not scoliosis |
| 16 | Webster et al., 2021 | Upper limb rehabilitation interventions using virtual reality for people with multiple sclerosis | R2 | Multiple sclerosis population |
| 17 | Hao et al., 2021 | Effects of virtual reality-based telerehabilitation for stroke patients | R2 | Stroke patients only |
| 18 | Gooch et al., 2024 | Behavior change approaches in digital technology-based physical rehabilitation interventions following stroke | R2 | Stroke rehabilitation focus |
| 19 | Anar, 2016 | The effectiveness of home-based exercise programs for low back pain patients | R2 | General low back pain, scoliosis not specified |
| 20 | Cornelius et al., 2025 | Wearable Technology, Smart Home Systems, and Mobile Apps for Dementia Care | R2 | Dementia care population |
| 21 | Hinman et al., 2020 | Telerehabilitation for knee osteoarthritis: a systematic review | R2 | Osteoarthritis patients |
| 22 | Neviaser & Neviaser, 2011 | Adhesive capsulitis of the shoulder | R2 | Shoulder condition, not spinal |
| 23 | Dong et al., 2024 | Revision Surgery After Spinal Fusion in Adolescent Idiopathic Scoliosis | R3 | Purely surgical study, no digital rehabilitation component |
| 24 | Valic et al., 2020 | Malpositioned pedicle screw in spine deformity surgery endangering the aorta | R3 | Surgical complication case series |
| 25 | Kuklo et al., 2007 | Surgical revision rates of hooks versus hybrid versus screws for adolescent idiopathic scoliosis | R3 | Surgical technique comparison, no digital intervention |
| 26 | Holewijn et al., 2017 | A novel spinal implant for fusionless scoliosis correction: biomechanical analysis | R3 | Biomechanical study of surgical device |
| 27 | Baldwin et al., 2020 | Does intraoperative CT navigation increase the accuracy of pedicle screw placement | R3 | Intraoperative navigation only, not rehabilitation |
| 28 | Kwan et al., 2016 | Accuracy and safety of pedicle screw placement in adolescent idiopathic scoliosis patients | R3 | Surgical technique study |
| 29 | Radomska et al., 2025 | Use of Monopolar Coagulation During Surgery for Advanced Scoliosis in Patients with Cochlear Implants | R3 | Surgical technique case report |
| 30 | Trobisch et al., 2010 | Idiopathic scoliosis surgical treatment outcomes | R3 | Traditional exercise only, no digital component |
| 31 | Kontodimopoulos et al., 2018 | Children's and parents' perspectives of health-related quality of life in newly diagnosed scoliosis | R3 | Cross-sectional survey, no intervention |
| 32 | Makino et al., 2019 | Health-related quality of life and postural changes in female adolescents with back pain in AIS | R3 | Observational study, no digital intervention |
| 33 | Willner & Uden, 1982 | A prospective prevalence study of scoliosis in Southern Sweden | R3 | Epidemiological study only |
| 34 | Hoashi et al., 2013 | Adolescent scoliosis classification and treatment | R3 | Review of traditional treatments only |
| 35 | Huang et al., 2024 | Evaluation of the prevalence of adolescent scoliosis and its associated factors: a cross-sectional study | R4 | Prevalence study only, no intervention outcomes |
| 36 | Tahirbegolli et al., 2021 | Factors affecting the prevalence of idiopathic scoliosis among children | R4 | Risk factor analysis, no treatment outcomes |
| 37 | Almahmoud et al., 2023 | Overview of adolescent idiopathic scoliosis and associated factors: a scoping review | R1 | Scoping review |
| **Update (May 12, 2026)** | | | | |
| 38 | Zhu et al., 2025 | Effect of exercise therapy on adolescent idiopathic scoliosis in mild to moderate: a systematic review and network meta-analysis | R1 | Network meta-analysis; not an original intervention study |
| 39 | Takinacı et al., 2025 | Enhancing Home-Based Exercise Therapy with Telerehabilitation in Mild Adolescent Idiopathic Scoliosis: A Randomized Controlled Trial | R3 | Telerehabilitation used solely as a delivery mode without a qualifying digital health intervention component |
| 40 | Wong et al., 2026 | Evaluating the Effectiveness of sEMG Biofeedback for Posture Training and Scoliosis Management. | R3 | Exists as a monitoring tool |
| 41 | Suresh et., 2025 | Digital twin for the analog scoliometer: advancements in telehealth for paediatric spine deformity care | R4 | No quantitative clinical outcome data reported |

**Reason Codes:**

R1 = Study design not meeting inclusion criteria (including systematic reviews, scoping reviews, meta-analyses, narrative reviews, clinical guidelines, study protocols, conference abstracts, case reports, and case series)

R2 = Study population not meeting inclusion criteria (participants not diagnosed with scoliosis, or mixed populations in which results for scoliosis patients were not reported separately)

R3 = Absence of a qualifying digital health intervention component (including purely surgical studies without postoperative digital rehabilitation, studies in which digital technology served solely as a passive delivery medium or monitoring tool without constituting a therapeutic intervention, and observational or epidemiological studies without any intervention)

R4 = Insufficient quantitative outcome data (studies that did not report extractable quantitative clinical outcome measures, including purely qualitative designs, descriptive surveys, and prevalence studies)

**Note:** A total of 54 studies underwent full-text assessment during the systematic review process, comprising 47 studies from database searches (including 5 identified through the updated search conducted on May 12, 2026) and 7 studies identified through citation searching. Of these, 13 studies met the inclusion criteria and were included in the final analysis. The remaining 41 studies were excluded for the reasons specified above. Where a study met more than one exclusion criterion, it was assigned to the criterion with the highest priority in the following prespecified order: study design (R1), study population (R2), intervention characteristics (R3), and outcome reporting (R4). This supplementary table provides transparency in the study selection process and adheres to PRISMA 2020 reporting guidelines.
