# Supplementary material for: Application of Digital Health Technologies in Scoliosis Rehabilitation: Systematic Review Based on the Technology Classification Framework
Source: J Med Internet Res. 2026 Aug 28;28:e91461. doi: 10.2196/91461 (PMC13524367; doi:10.2196/91461)
Supplement: Supplementary file 5 [file jmir-v28-e91461-s003.docx]

**Quality Assessment of Included Studies**

The Template for Intervention Description and Replication (TIDieR) checklist for assessing intervention reporting completeness (n=13).

Table S1. TIDieR Checklist for Intervention Reporting (n=13)

| Study (Author, Year) | Item 1 Name | Item 2 Why | Item 3 What (materials) | Item 4 What (procedures) | Item 5 Who provided | Item 6 How | Item 7 Where | Item 8 When and how much | Item 9 Tailoring | Item 10 Modifications | Item 11 How well (planned) | Item 12 How well (actual) | Reporting rate |
| --- | --- | --- | --- | --- | --- | --- | --- | --- | --- | --- | --- | --- | --- |
| Wan et al. (2024) [34] | ✓ | ✓ | ✓ | ✓ | ✓ | ✓ | ✓ | P | ✓ | ✓ | P | P | 9/12 |
| Andrade et al. (2024) [17] | ✓ | ✓ | ✓ | ✓ | ✓ | ✓ | ✓ | ✓ | ✓ | ✓ | ✓ | P | 11/12 |
| Dursun et al. (2024) [35] | ✓ | ✓ | ✓ | ✓ | ✓ | ✓ | ✓ | ✓ | ✓ | ✓ | P | ✓ | 11/12 |
| Fishman (2021) [36] | ✓ | ✓ | ✓ | ✓ | ✓ | ✓ | ✓ | ✓ | ✓ | ✗ | ✗ | P | 9/12 |
| Kisa et al. (2024) [37] | ✓ | ✓ | ✓ | ✓ | ✓ | ✓ | ✓ | ✓ | ✓ | ✓ | P | P | 10/12 |
| Lau et al. (2021) [38] | ✓ | ✓ | ✓ | ✓ | ✓ | ✓ | ✓ | ✓ | ✗ | ✓ | ✓ | ✓ | 11/12 |
| Tombak et al. (2024) [39] | ✓ | ✓ | ✓ | ✓ | ✓ | ✓ | ✓ | ✓ | ✓ | ✓ | P | ✓ | 11/12 |
| Zapata et al. (2015) [40] | ✓ | ✓ | ✓ | ✓ | ✓ | ✓ | ✓ | ✓ | P | ✓ | ✗ | P | 9/12 |
| Moraes et al. (2022) [41] | ✓ | ✓ | ✓ | ✓ | ✓ | ✓ | ✓ | P | ✓ | ✓ | ✓ | ✓ | 11/12 |
| Nam et al. (2023) [42] | ✓ | ✓ | ✓ | ✓ | ✓ | ✓ | P | P | ✓ | ✓ | ✓ | ✗ | 9/12 |
| Ozger et al. (2025) [43] | ✓ | ✓ | ✓ | ✓ | ✓ | ✓ | ✓ | ✓ | P | ✓ | ✓ | ✓ | 11/12 |
| Yuan et al. (2025) [44] | ✓ | ✓ | ✓ | ✓ | ✓ | ✓ | ✓ | ✓ | ✓ | ✓ | ✓ | ✓ | 12/12 |
| Chen et al. (2026) [45] | ✓ | ✓ | ✓ | ✓ | ✓ | ✓ | ✓ | ✓ | ✓ | P | ✓ | ✓ | 11/12 |

The TIDieR checklist consists of 12 items evaluating the completeness of intervention reporting: (Item 1) brief name of the intervention; (Item 2) rationale, theory, or goal; (Item 3) physical or informational materials used; (Item 4) detailed description of procedures and activities; (Item 5) provider characteristics and qualifications; (Item 6) modes of delivery; (Item 7) type of location where intervention occurred; (Item 8) number of sessions, frequency, duration, and intensity; (Item 9) whether and how the intervention was tailored to individuals; (Item 10) whether and how the intervention was modified during the study; (Item 11) assessment of intervention fidelity or adherence to protocol; and (Item 12) assessment of participant adherence or engagement. Each item was rated as "✓" (adequately reported), "P" (partially reported), or "✗" (not reported or inadequately reported) based on the information provided in the published manuscripts. The reporting rate represents the proportion of items adequately reported out of 12.

References:

17. Mantelatto Andrade R, Gomes Santana B, Verttú Schmidt A, et al. Effect of traditional rehabilitation programme versus telerehabilitation in adolescents with idiopathic scoliosis during the COVID-19 pandemic: a cohort study. J Rehabil Med. Feb 26, 2024;56:jrm5343. [doi: 10.2340/jrm.v56.5343] [Medline: 38407430]

34. Wan FKW, Mak ATH, Chung CWY, Yip JYW. Development of a motion-based video game for postural training: a feasibility study on older adults with adult degenerative scoliosis. IEEE Trans Neural Syst Rehabil Eng. 2024;32:2106-2113. [doi: 10.1109/TNSRE.2024.3398029] [Medline: 38717877]

35. Manzak Dursun AS, Ozyilmaz S, Ucgun H, Elmadag NM. The effect of pilates-based exercise applied with hybrid telerehabilitation method in children with adolescent idiopathic scoliosis: a randomized clinical trial. Eur J Pediatr. Feb 2024;183(2):759-767. [doi: 10.1007/s00431-023-05340-2] [Medline: 37993666]

36. Fishman LM. Isometric yoga-like maneuvers improve adolescent idiopathic scoliosis-a nonrandomized control trial. Glob Adv Health Med. 2021;10:2164956120988259. [doi: 10.1177/2164956120988259] [Medline: 33717658]

37. Kisa EP, Tarakcı E, Leblebici G, Ozdincler AR, Kasapcopur O. Exercise programs for scoliosis in juvenile idiopathic arthritis: a randomized-controlled study. Rheumatol Int. Mar 2024;44(3):441-449. [doi: 10.1007/s00296-023-05439-7] [Medline: 37668835]

38. Lau RWL, Cheuk KY, Ng BKW, et al. Effects of a home-based exercise intervention (E-Fit) on bone density, muscle function, and quality of life in girls with adolescent idiopathic scoliosis (AIS): a pilot randomized controlled trial. Int J Environ Res Public Health. Oct 17, 2021;18(20):10899. [doi: 10.3390/ijerph182010899] [Medline: 34682668]

39. Tombak K, Yüksel İ, Ozsoy U, Yıldırım Y, Karaşin S. A comparison of the effects of supervised versus home schroth exercise programs with adolescent idiopathic scoliosis. Children (Basel). Mar 17, 2024;11(3):354. [doi: 10.3390/children11030354] [Medline: 38539389]

40. Zapata KA, Wang-Price SS, Sucato DJ, Thompson M, Trudelle-Jackson E, Lovelace-Chandler V. Spinal stabilization exercise effectiveness for low back pain in adolescent idiopathic scoliosis: a randomized trial. Pediatr Phys Ther. 2015;27(4):396-402. [doi: 10.1097/PEP.0000000000000174] [Medline: 26397085]

41. Moraes ÍA, Cardoso A, Soares AB, de Oliveira DAR, Júnior EL. Combining immersive virtual reality and postural therapy for scoliosis treatment and prevention in children. Res Biomed Eng. 2022;38(3):1003-1016. [doi: 10.1007/s42600-022-00235-x]

42. Nam YG, Park SY, Kwon BS. Rehabilitation exercise using a smart-bar device with augmented reality guide function. Int J Precis Eng Manuf. Jul 2023;24(7):1263-1270. [doi: 10.1007/s12541-023-00815-6]

43. Ozger EY, Mustafaoglu R, Nimetoglu B, Akgul T. Efficacy of virtual reality-based rehabilitation following posterior fusion in adolescent idiopathic scoliosis: a single-blind randomized trial. Eur Spine J. Jan 2026;35(1):36-45. [doi: 10.1007/s00586-025-09501-9] [Medline: 41201608]

44. Yuan W, Shi W, Chen L, et al. Digital physiotherapeutic scoliosis-specific exercises for adolescent idiopathic scoliosis: a randomized clinical trial. JAMA Netw Open. Feb 3, 2025;8(2):e2459929. [doi: 10.1001/jamanetworkopen.2024.59929] [Medline: 39964686]

45. Chen T, Zhou H, Chen Q, et al. Effects of bracing combined with tele-rehabilitation-guided family physiotherapeutic scoliosis-specific exercises on adolescent idiopathic scoliosis. Orthop Surg. Mar 2026;18(3):511-522. [doi: 10.1111/os.70265] [Medline: 41615191]
