# Supplementary material for: Application of Digital Health Technologies in Scoliosis Rehabilitation: Systematic Review Based on the Technology Classification Framework
Source: J Med Internet Res. 2026 Aug 28;28:e91461. doi: 10.2196/91461 (PMC13524367; doi:10.2196/91461)
Supplement: Supplementary file 6 [file jmir-v28-e91461-s004.docx]

**Table S1. Extracted Outcome Data Supporting the Direction-of-Effect Synthesis.**

| **Author [Ref]** | **Outcome / Instrument** | **Intervention Group**  **(post-value or change, mean ± SD)** | **Control Group**  **(post-value or change, mean ± SD)** | **Between-Group Difference (P value)** | **Direction** |
| --- | --- | --- | --- | --- | --- |
| **1. Clinical Outcomes** | | | | | |
| **1.1 Cobb Angle (MCIDᵃ = 3.5°)** | | | | | |
| **Andrade et al [17]** | Major curve Cobb angle (°) | Post (6 mo): 34.6 ± 7.7°  Change: −2.4° (within-group P = .027) | Post (6 mo): 34.1 ± 7.6°  Change: −4.9° (within-group P = .019) | No significant between-group difference; both groups improved. Traditional group showed larger effect (d = 0.74 vs d = 0.33). Point estimate favours control group; direction coded as unfavorable (↓). | **↓** |
| **Dursun et al [35]** | Primary curve Cobb angle (°) | Baseline: 37.50 ± 7.37°  Post (12 wk): 32.43 ± 7.06°  Change: −5.06 ± 4.32° | Baseline: 31.60 ± 6.08°  Post (12 wk): 30.57 ± 6.64°  Change: −1.02 ± 3.85° | INTᵇ superior by 4.04°; time × group P = .011, ηp² = 0.205, d = 0.59. | **↑** |
| **Fishman [36]** | Cobb angle (°)  (thoracolumbar & thoracic; median) | Thoracolumbar: −9.2° (95% CI −11.8 to −6.6; P < .001)  Thoracic: −7.1° (95% CI −13.1 to −1.2; P = .022) | Lumbar: +5.4° worsening (95% CI 1.7 to 9.0; P = .008)  Thoracic: +9.3° worsening (95% CI 4.5 to 14.6; P = .006) | Thoracolumbar: INT superior; Mann-Whitney U = 159.0, P = .030.  Thoracic: P = .562 (not significant). | **↑** |
| **Kisa et al [37]** | Cobb angle (°) | Baseline: 16.96 ± 3.12°  Post (24 wk): 9.96 ± 2.80°  Change: −7.0° | Baseline: 16.48 ± 5.73°  Post (24 wk): 13.48 ± 4.55°  Change: −3.0° | INT superior by 4.0°; P < .001, d = 2.36. | **↑** |
| **Lau et al. [38]** | Cobb angle (°) | angle (°)Reported but unevaluable: accelerometer synchronisation failure (~15% sync rate) precluded confirmation of treatment exposure; risk of insufficient treatment cannot be excluded. | — | Indeterminate | **○** |
| **Yuan et al. [44]** | Major curve Cobb angle (°)  (intention-to-treat analysis) | Baseline: 15.78° (95% CI 14.70–16.86)  Post (6 mo): 7.42°  Change: −8.36° (95% CI −9.73 to −6.99)  Improvement rate: 76.6% | Baseline: 16.03° (95% CI 14.90–17.16)  Post (6 mo): 11.91°  Change: −4.13° (95% CI −5.39 to −2.86)  Improvement rate: 45.3% | Between-group: −4.23° (95% CI −6.08 to −2.39); P < .001.  Improvement rate difference: P = .001. | **↑** |
| **Chen et al. [45]** | Major curve Cobb angle (°) (24-mo) | Baseline: 28.6 ± 5.6°  Post (24 mo): 21.8 ± 5.8°  Change: −6.8°  Improvement rate: 70.6% | Baseline: 28.9 ± 6.3°  Post (24 mo): 25.4 ± 6.6°  Change: −3.5°  Improvement rate: 57.6% | 12-mo: P = .029; 24-mo: P = .021.  Subgroup (≥4 sessions/week): −6.8° vs −4.6°, P < .001. | **↑** |
| **1.2 ATRᶜ** | | | | | |
| **Dursun et al. [35]** | ATR (thoracic & lumbar; °) | Thoracic: 7.12 ± 5.52 → 6.40 ± 4.95° (Δ = −0.71°; P = .031)  Lumbar: 5.00 ± 4.13 → 4.37 ± 3.75° (Δ = −0.62°; P = .046) | Thoracic: 5.86 ± 4.73 → 4.92 ± 4.07° (Δ = −0.93°; P = .011)  Lumbar: 6.60 ± 3.33 → 5.78 ± 3.18° (Δ = −0.81°; P = .011) | No significant between-group difference.  Thoracic: P = .621; Lumbar: P = .639. | **○** |
| **Kisa et al. [37]** | ATR (°) | Baseline: 6.82 ± 1.48°  Post (24 wk): 2.72 ± 1.86°  Change: −4.1° | Baseline: 6.56 ± 1.93°  Post (24 wk): 4.36 ± 2.34°  Change: −2.2° | INT superior by 1.9°; P = .007, d = 2.43. | **↑** |
| **Tombak et al. [39]** | ATR (thoracic & lumbar; °) | Thoracic: 6.84 → 3.56° (Δ = −3.28°; P < .001)  Lumbar: 5.95 → 3.26° (Δ = −2.69°; P < .001) | Thoracic: 6.11 → 3.44° (Δ = −2.67°; P = .001)  Lumbar: 5.39 → 3.17° (Δ = −2.22°; P < .001) | No significant between-group difference.  Thoracic interaction: P = .445. | **○** |
| **Yuan et al. [44]** | ATR (°) | Change: −0.87° | Change: −0.49° | No significant between-group difference; P = .64. | **○** |
| **Chen et al. [45]** | ATR (°; 24-mo) | Baseline: 10.9 ± 2.8°  Post (24 mo): 6.9 ± 1.9°  Change: −4.0° | Baseline: 10.7 ± 3.2°  Post (24 mo): 8.7 ± 2.9°  Change: −2.0° | INT superior by 1.8°; P = .005. | **↑** |
| **1.3 Spinal Flexibility** | | | | | |
| **Moraes et al. [41]** | Standing tolerance (s); CoPᵈ displacement (cm) | Standing: 146.82 ± 90.57 → 158.91 ± 55.30 s  Right-hand displacement: 10.47 ± 5.17 → 7.08 ± 5.15 cm  Sitting CoP: 9.06 ± 6.08 → 6.94 ± 3.55 cm | Standing: 81.73 ± 33.33 → 94.18 ± 28.11 s  Right-hand displacement: 10.70 ± 5.97 → 12.37 ± 6.19 cm  Sitting CoP: 9.07 ± 5.40 → 12.54 ± 4.69 cm | Standing tolerance: P = .001; right-hand displacement: P = .021; sitting CoP: P = .002. All INT superior. | **↑** |
| **Ozger et al. [43]** | Modified Schober test (cm) | Baseline: 19.29 ± 1.54 cm  Post (6 wk): 20.71 ± 1.49 cm (within-group P < .001) | Baseline: 19.50 ± 1.60 cm  Post (6 wk): 19.50 ± 1.60 cm (no change; P > .05) | INT superior; P = .04. | **↑** |
| **2. Functional Outcomes** | | | | | |
| **2.1 Respiratory Function** | | | | | |
| **Dursun et al. [35]** | MIPᵉ, MEPᶠ (cmH₂O); PEF%ᵍ, FVC%ʰ, FEV1%ⁱ | MIP: +25.43 ± 8.69 (P < .001)  MEP: +28.50 ± 5.58 (P < .001)  PEF%: +13.87 ± 12.85 (P = .001)  FVC%: +1.87 (P = .141); FEV1%: +3.43 (P = .006) | MIP: +13.94 ± 13.12 (P = .001)  MEP: +17.94 ± 15.15 (P < .001)  PEF%: +4.08 ± 10.13 (P = .141)  FVC%: +2.14 (P = .239); FEV1%: +0.99 (P = .395) | MIP: INT superior; P = .011, ηp² = 0.203.  MEP: INT superior; P = .034, ηp² = 0.146.  PEF%: INT superior; P = .026.  FVC% and FEV1%: not significant. | **↑** |
| **Kisa et al. [37]** | FVC%, FEV1% (% predicted) | FVC%: 87.40 ± 7.62 → 93.76 ± 7.44 (Δ = +6.36%; P < .001)  FEV1%: 91.32 ± 10.23 → 99.16 ± 6.65 (Δ = +7.84%; P < .001) | FVC%: 86.44 ± 5.89 → 88.28 ± 5.94 (Δ = +1.84%; P = .004)  FEV1%: 91.40 ± 8.10 → 93.96 ± 6.80 (Δ = +2.56%; P = .094) | FVC%: INT superior; P = .020.  FEV1%: INT superior; P = .035. | **↑** |
| **Ozger et al. [43]** | MIP (cmH₂O) | Baseline: 63.21 cmH₂O  Post (6 wk): 83.36 cmH₂O (Δ = +20.64; P < .001) | Baseline: 64.57 cmH₂O  Post (6 wk): 69.79 cmH₂O (Δ = +5.21; P = .119) | INT superior; P = .02. | **↑** |
| **2.2 Postural Control / Gait** | | | | | |
| **Moraes et al. [41]** | Standing tolerance (s); sitting CoPᵈ displacement (cm) | Standing: 146.82 ± 90.57 → 158.91 ± 55.30 s  Sitting CoP: 9.06 ± 6.08 → 6.94 ± 3.55 cm  Faster self-correction after fatigue | Standing: 81.73 ± 33.33 → 94.18 ± 28.11 s  Sitting CoP: 9.07 ± 5.40 → 12.54 ± 4.69 cm | Standing tolerance: P = .001; sitting CoP: P = .002. INT superior in both. | **↑** |
| **Ozger et al. [43]** | Spinal balance (SVAʲ, PTᵏ); kinesiophobia (TSKˡ) | PT: 17.81° → 11.02° (Δ = −6.79°; P < .001)  SVA: within-group P = .01  TSK: 41.86 → 38.48 (Δ = −3.38; P = .01) | PT: no significant change (P = .88)  SVA: no significant change (P = .33)  TSK: 37.36 → 36.86 (Δ = −0.50; P = .73) | PT: between-group P = .19 (NS). SVA: between-group P = .62 (NS). TSK: between-group P = .69 (NS).  VRᵐ group: significant within-group improvement; home exercise group: no improvement. | **↑** |
| **Yuan et al. [44]** | Pelvic tilt angle throughout gait cycle (°) | All 4 gait time points: superior pelvic tilt alignment. Improvement range: 0.76°–1.23° | Inferior pelvic tilt alignment vs INT across all 4 gait parameters | Stance phase: min −1.23° (P = .02), max −0.76° (P = .02).  Swing phase: min −1.06° (P = .02), max −0.84° (P = .04). All INT superior. | **↑** |
| **3. Patient-Reported Outcomes** | | | | | |
| **3.1 Health-Related Quality of Life (SRS-22^n^)** | | | | | |
| **Tombak et al. [39]** | SRS-22 (total score; 1–5) | SSEG^o^: 4.01 → 4.22 (Δ = +0.21; P < .001) | HSEG^p^: 3.91 → 4.16 (Δ = +0.25; P < .001) | Both groups improved significantly; no between-group difference; P = .622. | **○** |
| **Lau et al. [38]** | SRS-22 (self-image; total) | Self-image: 3.78 ± 0.59 → 3.82 ± 0.56 (time × group P = .066)  Total: time × group P = .086 | No significant improvement | Marginal trend favouring INT in self-image (P = .066) and total score (P = .086). | **↑** |
| **Ozger et al. [43]** | SRS-22 (total score; 1–5) | 3.66 → 3.89 (Δ = +0.23; within-group P = .01) | 3.98 → 4.04 (Δ = +0.06; within-group P = .08) | VR group: significant within-group improvement; home exercise group: no significant improvement; between-group: P = .73 (NS). | **↑** |
| **Chen et al. [45]** | SRS-22 (all 5 domains; 24-mo) | All 5 domains: significant at 24 mo (all P < .05).  Function & satisfaction: significant at 12 mo. | All 5 domains: significant at 24 mo (all P < .05).  Function & satisfaction: not significant at 12 mo. | Earlier onset of clinically meaningful improvement in function and satisfaction domains in INT group at 12 mo (P < .05). | **↑** |
| **3.2 Pain** | | | | | |
| **Kisa et al. [37]** | Joint pain (NRS^q^, 0–10) | Baseline: 4.60 ± 1.29  Post (24 wk): 1.48 ± 1.12  Change: −3.12 pts | Baseline: 4.04 ± 1.12  Post (24 wk): 2.48 ± 1.08  Change: −1.56 pts | INT superior by 1.56 pts; P < .001. | **↑** |
| **Zapata et al. [40]** | Low back pain (NRS, 0–10) | Supervised + DVD: 5.4 → 1.5 (Δ = −3.9 pts) | DVD-only: 5.4 → 3.4 (Δ = −2.2 pts) | Supervised group superior by 1.7 pts; P = .005. | **↓** |
| **3.3 Body Image (WRVAS^r^)** | | | | | |
| **Kisa et al. [37]** | WRVAS (0–35; higher = worse) | Baseline: 15.60 ± 4.72  Post (24 wk): 9.56 ± 2.43  Change: −6.04 pts | Baseline: 16.56 ± 4.62  Post (24 wk): 13.32 ± 3.82  Change: −3.24 pts | INT superior by 2.80 pts; P = .002, d = 1.60. | **↑** |
| **Tombak et al. [39]** | WRVAS (0–35) | SSEG: 17.58 → 13.84 (Δ = −3.74 pts; P < .001) | HSEG: 19.50 → 15.28 (Δ = −4.22 pts; P < .001) | Both groups improved significantly; no between-group difference; P = .680. | **○** |
| **4. Implementation Outcomes** | | | | | |
| **4.1 Adherence** | | | | | |
| **Fishman [36]** | Non-adherence rate (%; < 4 sessions/week) | Remote video group: 36.7% non-adherence | Clinic face-to-face: 20% non-adherence | Remote group had higher non-adherence than clinic group (P not reported). | **↓** |
| **Kisa et al. [37]** | Exercise compliance; WhatsApp monitoring | Dropout: 0% (25/25 completed)  WhatsApp daily monitoring: compliant | Dropout: 0% (25/25 completed) | Zero dropouts in both groups (100% completion in both arms); no between-group difference in completion rate. Direction coded as no clear direction (○). | **○** |
| **Lau et al. [38]** | Accelerometer sync rate (%) | Mean sync rate: ~15% (range: 0%–64%)  Majority failed to sync regularly | Not applicable (no wearable device assigned) | Digital adherence monitoring unsuccessful due to device synchronisation failures. | **○** |
| **Tombak et al. [39]** | Session compliance rate (%) | Supervised sessions: 100%; home component: 97.54% | Home-video component: 96.89% | Both groups showed high compliance. | **○** |
| **Zapata et al. [40]** | Session adherence rate (%) | Supervised + DVD: 95% | DVD-only: 67% | Supervised group significantly higher; P = .001. | **↓** |
| **Ozger et al. [43]** | Session completion (%); physical activity (steps/day) | VR: 100% session completion  Steps/day: 5,275 → 8,717 (Δ = +3,441; P < .001) | Home exercise: phone-confirmed  Steps/day: 3,343 → 3,819 (Δ = +476; P = .241) | Session completion: 100% (VR group). Physical activity: INT superior; P = .01. | **↑** |
| **Yuan et al. [44]** | Exercise frequency (sessions/week); duration (min/week) | Frequency: 5.30 ± 0.57 sessions/week  Duration: 179.44 ± 24.18 min/week; ≥6 days/week: 91.2% | Frequency: 4.63 ± 0.60 sessions/week  Duration: 147.84 ± 23.75 min/week; ≥6 days/week: 66.7% | Frequency: INT superior; P < .001.  Duration: INT superior; P < .001.  ≥6 days/week rate: P = .003. | **↑** |
| **Chen et al. [45]** | Session completion rate (%); ≥80% adherence rate (%) | Completion: 92.6% ± 5.9%; ≥80% adherence: 97.1% | Completion: 74.3% ± 12.8%; ≥80% adherence: 60.6% | Completion: INT superior; P < .001. ≥80% adherence: INT superior; P < .001. | **↑** |
| **4.2 Dropout/Loss-to-Follow-Up Rates** | | | | | |
| **Kisa et al. [37]** | Dropout rate (%) | 0% (25/25 completed) | 0% (25/25 completed) | Zero dropouts in both groups (0% vs. 0%); no between-group difference. Direction coded as no clear direction (○). | **○** |
| **Zapata et al. [40]** | Dropout rate (%) | Supervised + DVD: 11% | DVD-only: 35% | Unsupervised group had significantly higher dropout. | **↓** |
| **Yuan et al. [44]** | Dropout rate (%) | Digital care group: 10.9% (7/64) | Conventional: 15.6% (10/64) | Digital care group had lower dropout (P not reported). | **↑** |
| **Chen et al. [45]** | Dropout rate (%) | INT group: 5.9% (2/34) | Autonomous group: 15.2% (5/33) | INT group had lower dropout; P = .261 (NS). | **↑** |

↑ = favorable effect direction; ↓ = unfavorable effect direction; ○ = no clear effect direction. Blank cells indicate that the outcome was not assessed in that study. Direction of effect is reported regardless of statistical significance.

Feasibility studies (Wan et al. [34]; Nam et al. [42]) are excluded from this table as they lack a controlled comparator. All values are mean ± SD unless otherwise stated. Between-group differences reflect time × group interaction effects, independent t-tests, analysis of covariance, or Mann-Whitney U tests as reported in the source publications. Effect sizes (Cohen d) are extracted from source publications where reported.

ᵃ MCID: minimal clinically important difference.

ᵇ INT: intervention group.

ᶜ ATR: angle of trunk rotation.

ᵈ CoP: center of pressure.

ᵉ MIP: maximal inspiratory pressure.

ᶠ MEP: maximal expiratory pressure.

ᵍ PEF%: peak expiratory flow percentage predicted.

ʰ FVC%: forced vital capacity percentage predicted.

ⁱ FEV1%: forced expiratory volume in 1 second percentage predicted.

ʲ SVA: sagittal vertical axis.

ᵏ PT: pelvic tilt.

ˡ TSK: Tampa Scale of Kinesiophobia.

^m^ VR: virtual reality.

ⁿ MBQ: Modified Baecke Questionnaire.

^n^ SRS-22: Scoliosis Research Society 22-item questionnaire.

^o^ SSEG: supervised scoliosis exercise group (Tombak et al. [39]).

^p^ HSEG: home-based scoliosis exercise group (Tombak et al. [39]).

^q^ NRS: numerical rating scale.

^r^ WRVAS: Walter Reed Visual Assessment Scale.

**References**:

17. Mantelatto Andrade R, Gomes Santana B, Verttú Schmidt A, et al. Effect of traditional rehabilitation programme versus telerehabilitation in adolescents with idiopathic scoliosis during the COVID-19 pandemic: a cohort study. J Rehabil Med. Feb 26, 2024;56:jrm5343. [doi: 10.2340/jrm.v56.5343] [Medline: 38407430]

34. Wan FKW, Mak ATH, Chung CWY, Yip JYW. Development of a motion-based video game for postural training: a feasibility study on older adults with adult degenerative scoliosis. IEEE Trans Neural Syst Rehabil Eng. 2024;32:2106-2113. [doi: 10.1109/TNSRE.2024.3398029] [Medline: 38717877]

35. Manzak Dursun AS, Ozyilmaz S, Ucgun H, Elmadag NM. The effect of pilates-based exercise applied with hybrid telerehabilitation method in children with adolescent idiopathic scoliosis: a randomized clinical trial. Eur J Pediatr. Feb 2024;183(2):759-767. [doi: 10.1007/s00431-023-05340-2] [Medline: 37993666]

36. Fishman LM. Isometric yoga-like maneuvers improve adolescent idiopathic scoliosis-a nonrandomized control trial. Glob Adv Health Med. 2021;10:2164956120988259. [doi: 10.1177/2164956120988259] [Medline: 33717658]

37. Kisa EP, Tarakcı E, Leblebici G, Ozdincler AR, Kasapcopur O. Exercise programs for scoliosis in juvenile idiopathic arthritis: a randomized-controlled study. Rheumatol Int. Mar 2024;44(3):441-449. [doi: 10.1007/s00296-023-05439-7] [Medline: 37668835]

38. Lau RWL, Cheuk KY, Ng BKW, et al. Effects of a home-based exercise intervention (E-Fit) on bone density, muscle function, and quality of life in girls with adolescent idiopathic scoliosis (AIS): a pilot randomized controlled trial. Int J Environ Res Public Health. Oct 17, 2021;18(20):10899. [doi: 10.3390/ijerph182010899] [Medline: 34682668]

39. Tombak K, Yüksel İ, Ozsoy U, Yıldırım Y, Karaşin S. A comparison of the effects of supervised versus home schroth exercise programs with adolescent idiopathic scoliosis. Children (Basel). Mar 17, 2024;11(3):354. [doi: 10.3390/children11030354] [Medline: 38539389]

40. Zapata KA, Wang-Price SS, Sucato DJ, Thompson M, Trudelle-Jackson E, Lovelace-Chandler V. Spinal stabilization exercise effectiveness for low back pain in adolescent idiopathic scoliosis: a randomized trial. Pediatr Phys Ther. 2015;27(4):396-402. [doi: 10.1097/PEP.0000000000000174] [Medline: 26397085]

41. Moraes ÍA, Cardoso A, Soares AB, de Oliveira DAR, Júnior EL. Combining immersive virtual reality and postural therapy for scoliosis treatment and prevention in children. Res Biomed Eng. 2022;38(3):1003-1016. [doi: 10.1007/s42600-022-00235-x]

42. Nam YG, Park SY, Kwon BS. Rehabilitation exercise using a smart-bar device with augmented reality guide function. Int J Precis Eng Manuf. Jul 2023;24(7):1263-1270. [doi: 10.1007/s12541-023-00815-6]

43. Ozger EY, Mustafaoglu R, Nimetoglu B, Akgul T. Efficacy of virtual reality-based rehabilitation following posterior fusion in adolescent idiopathic scoliosis: a single-blind randomized trial. Eur Spine J. Jan 2026;35(1):36-45. [doi: 10.1007/s00586-025-09501-9] [Medline: 41201608]

44. Yuan W, Shi W, Chen L, et al. Digital physiotherapeutic scoliosis-specific exercises for adolescent idiopathic scoliosis: a randomized clinical trial. JAMA Netw Open. Feb 3, 2025;8(2):e2459929. [doi: 10.1001/jamanetworkopen.2024.59929] [Medline: 39964686]

45. Chen T, Zhou H, Chen Q, et al. Effects of bracing combined with tele-rehabilitation-guided family physiotherapeutic scoliosis-specific exercises on adolescent idiopathic scoliosis. Orthop Surg. Mar 2026;18(3):511-522. [doi: 10.1111/os.70265] [Medline: 41615191]
