# Supplementary material for: Application of Digital Health Technologies in Scoliosis Rehabilitation: Systematic Review Based on the Technology Classification Framework
Source: J Med Internet Res. 2026 Aug 28;28:e91461. doi: 10.2196/91461 (PMC13524367; doi:10.2196/91461)
Supplement: Supplementary file 7 [file jmir-v28-e91461-s005.docx]

**Multimedia Appendix 5**

Direction-of-effect synthesis, subgroup analyses, sensitivity analysis, and GRADE certainty-of-evidence assessment.

**Contents**

Table S1. Direction-of-Effect Summary of Digital Health Interventions Across Outcome Indicators.	2

Table S2. Direction-of-Effect Summary of Digital Health Interventions by Technical Pathway.	2

Table S3. Direction-of-Effect Summary of Digital Health Interventions by Supervision Model.	4

Table S4. Direction-of-Effect Summary of Digital Health Interventions by Comparator Type.	6

Table S5. Direction-of-Effect Summary of Digital Health Interventions by Intervention Dose.	8

Table S6. Direction-of-Effect Summary of Digital Health Interventions by Bracing Co-intervention Status.	9

Table S7. Sensitivity Analysis: Direction-of-Effect Vote Counting Restricted to Studies at Low or Some Concerns Risk of Bias.	10

Table S8. Certainty of the Evidence (GRADE) for Each Outcome in the Direction-of-Effect Synthesis.	11

Table S1. Direction-of-Effect Summary of Digital Health Interventions Across Outcome Indicators.

| **Author [Ref]** | **Clinical Outcomes** | | | **Functional Outcomes** | | **Patient-Reported Outcomes** | | | **Implementation Outcomes** | |
| --- | --- | --- | --- | --- | --- | --- | --- | --- | --- | --- |
|  | **Cobb angle** | **ATR^a^** | **Spinal flexibility** | **Respiratory function** | **Postural control / Gait** | **Health-related quality of life** | **Pain** | **Body image** | **Adherence** | **Dropout/Loss-to-Follow-Up Rates** |
| Andrade et al. [17] | ↓ |  |  |  |  |  |  |  |  |  |
| Dursun et al. [35] | ↑ | ○ |  | ↑ |  |  |  |  |  |  |
| Fishman [36] | ↑ |  |  |  |  |  |  |  | ↓ |  |
| Kisa et al. [37] | ↑ | ↑ |  | ↑ |  |  | ↑ | ↑ | ○ | ○ |
| Lau et al. [38] | ○ |  |  |  |  | ↑ |  |  | ○ |  |
| Tombak et al. [39] |  | ○ |  |  |  | ○ |  | ○ | ○ |  |
| Zapata et al. [40] |  |  |  |  |  |  | ↓ |  | ↓ | ↓ |
| Moraes et al. [41] |  |  | ↑ |  | ↑ |  |  |  |  |  |
| Ozger et al. [43] |  |  | ↑ | ↑ | ↑ | ↑ |  |  | ↑ |  |
| Yuan et al. [44] | ↑ | ○ |  |  | ↑ |  |  |  | ↑ | ↑ |
| Chen et al. [45] | ↑ | ↑ |  |  |  | ↑ |  |  | ↑ | ↑ |
| **Total, n** | **7** | **5** | **2** | **3** | **3** | **4** | **2** | **2** | **8** | **4** |
| Favorable effect direction, n (%) | 5 (71.4%) | 2 (40.0%) | 2 (100.0%) | 3 (100.0%) | 3 (100.0%) | 3 (75.0%) | 1 (50.0%) | 1 (50.0%) | 3 (37.5%) | 2 (50.0%) |
| Unfavorable effect direction, n (%) | 1 (14.3%) | 0 (0.0%) | 0 (0.0%) | 0 (0.0%) | 0 (0.0%) | 0 (0.0%) | 1 (50.0%) | 0 (0.0%) | 2 (25.0%) | 1 (25.0%) |
| No clear effect direction, n (%) | 1 (14.3%) | 3 (60.0%) | 0 (0.0%) | 0 (0.0%) | 0 (0.0%) | 1 (25.0%) | 0 (0.0%) | 1 (50.0%) | 3 (37.5%) | 1 (25.0%) |

↑ = favorable effect direction; ↓ = unfavorable effect direction; ○ = no clear effect direction. Blank cells indicate that the outcome was not assessed in that study. Direction of effect is reported regardless of statistical significance. The two feasibility studies (Wan et al. [34]; Nam et al. [42]) are excluded from this synthesis owing to the absence of a controlled comparator.

^a^ATR: angle of trunk rotation.

Table S2. Direction-of-Effect Summary of Digital Health Interventions by Technical Pathway.

| **Author [Ref]** | **Clinical Outcomes** | | | **Functional Outcomes** | | **Patient-Reported Outcomes** | | | **Implementation Outcomes** | |
| --- | --- | --- | --- | --- | --- | --- | --- | --- | --- | --- |
|  | **Cobb angle** | **ATR^a^** | **Spinal flexibility** | **Respiratory function** | **Postural control / Gait** | **Health-related quality of life** | **Pain** | **Body image** | **Adherence** | **Dropout/Loss-to-Follow-Up Rates** |
| **Synchronous Telerehabilitation Technologies (n = 5)** | | | | | | | | | | |
| Andrade et al. [17] | ↓ |  |  |  |  |  |  |  |  |  |
| Dursun et al. [35] | ↑ | ○ |  | ↑ |  |  |  |  |  |  |
| Fishman [36] | ↑ |  |  |  |  |  |  |  | ↓ |  |
| Kisa et al. [37] | ↑ | ↑ |  | ↑ |  |  | ↑ | ↑ | ○ | ○ |
| Chen et al. [45] | ↑ | ↑ |  |  |  | ↑ |  |  | ↑ | ↑ |
| **Total, n** | **5** | **3** | **–** | **2** | **–** | **1** | **1** | **1** | **3** | **2** |
| Favorable effect direction, n (%) | 4 (75.0%) | 2 (66.7%) | – | 2 (100.0%) | – | 1 (100.0%) | 1 (100.0%) | 1 (100.0%) | 1 (33.3%) | 1 (50.0%) |
| Unfavorable effect direction, n (%) | 1 (25.0%) | 0 (0.0%) | – | 0 (0.0%) | – | 0 (0.0%) | 0 (0.0%) | 0 (0.0%) | 1 (33.3%) | 0 (0.0%) |
| No clear effect direction, n (%) | 0 (0.0%) | 1 (33.3%) | – | 0 (0.0%) | – | 0 (0.0%) | 0 (0.0%) | 0 (0.0%) | 1 (33.3%) | 1 (50.0%) |
| **Asynchronous Digital Educational Tools (n = 3)** | | | | | | | | | | |
| Lau et al. [38] | ○ |  |  |  |  | ↑ |  |  | ○ |  |
| Tombak et al. [39] |  | ○ |  |  |  | ○ |  | ○ | ○ |  |
| Zapata et al. [40] |  |  |  |  |  |  | ↓ |  | ↓ | ↓ |
| **Total, n** | **1** | **1** | **–** | **–** | **–** | **2** | **1** | **1** | **3** | **1** |
| Favorable effect direction, n (%) | 0 (0.0%) | 0 (0.0%) | – | – | – | 1 (50.0%) | 0 (0.0%) | 0 (0.0%) | 0 (0.0%) | 0 (0.0%) |
| Unfavorable effect direction, n (%) | 0 (0.0%) | 0 (0.0%) | – | – | – | 0 (0.0%) | 1 (100.0%) | 0 (0.0%) | 1 (33.3%) | 1 (100.0%) |
| No clear effect direction, n (%) | 1 (100.0%) | 1 (100.0%) | – | – | – | 1 (50.0%) | 0 (0.0%) | 1 (100.0%) | 2 (66.7%) | 0 (0.0%) |
| **Immersive VR^b^/AR^c^ Technologies (n = 2)** | | | | | | | | | | |
| Moraes et al. [41] |  |  | ↑ |  | ↑ |  |  |  |  |  |
| Ozger et al. [43] |  |  | ↑ | ↑ | ↑ | ↑ |  |  | ↑ |  |
| **Total, n** | **–** | **–** | **2** | **1** | **2** | **1** | **–** | **–** | **1** | **–** |
| Favorable effect direction, n (%) | – | – | 2 (100.0%) | 1 (100.0%) | 2 (100.0%) | 1 (100.0%) | – | – | 1 (100.0%) | – |
| Unfavorable effect direction, n (%) | – | – | 0 (0.0%) | 0 (0.0%) | 0 (0.0%) | 0 (0.0%) | – | – | 0 (0.0%) | – |
| No clear effect direction, n (%) | – | – | 0 (0.0%) | 0 (0.0%) | 0 (0.0%) | 0 (0.0%) | – | – | 0 (0.0%) | – |
| **Wearable Biosensing Technologies (n = 1)** | | | | | | | | | | |
| Lau et al. [38] | ○ |  |  |  |  | ↑ |  |  | ○ |  |
| **Total, n** | **1** | **–** | **–** | **–** | **–** | **1** | **–** | **–** | **1** | **–** |
| Favorable effect direction, n (%) | 0 (0.0%) | – | – | – | – | 1 (100.0%) | – | – | 0 (0.0%) | – |
| Unfavorable effect direction, n (%) | 0 (0.0%) | – | – | – | – | 0 (0.0%) | – | – | 0 (0.0%) | – |
| No clear effect direction, n (%) | 1 (100.0%) | – | – | – | – | 0 (0.0%) | – | – | 1 (100.0%) | – |
| **Integrated Digital Health Platforms (n = 1)** | | | | | | | | | | |
| Yuan et al. [44] | ↑ | ○ |  |  | ↑ |  |  |  | ↑ | ↑ |
| **Total, n** | **1** | **1** | **–** | **–** | **1** | **–** | **–** | **–** | **1** | **1** |
| Favorable effect direction, n (%) | 1 (100.0%) | 0 (0.0%) | – | – | 1 (100.0%) | – | – | – | 1 (100.0%) | 1 (100.0%) |
| Unfavorable effect direction, n (%) | 0 (0.0%) | 0 (0.0%) | – | – | 0 (0.0%) | – | – | – | 0 (0.0%) | 0 (0.0%) |
| No clear effect direction, n (%) | 0 (0.0%) | 1 (100.0%) | – | – | 0 (0.0%) | – | – | – | 0 (0.0%) | 0 (0.0%) |

↑ = favorable effect direction; ↓ = unfavorable effect direction; ○ = no clear effect direction. Blank cells indicate that the outcome was not assessed in that study. Direction of effect is reported regardless of statistical significance. Studies were stratified according to the five technical pathways described in Table 3 of the main text. One study employed both wearable biosensing and asynchronous digital education components concurrently and is therefore represented in two categories. The two feasibility studies (Wan et al. [34]; Nam et al. [42]) are excluded from this synthesis owing to the absence of a controlled comparator.

^a^ ATR: angle of trunk rotation.

^b^ VR: virtual reality.

^c^ AR: augmented reality.

Table S3. Direction-of-Effect Summary of Digital Health Interventions by Supervision Model.

| **Author [Ref]** | **Clinical Outcomes** | | | **Functional Outcomes** | | **Patient-Reported Outcomes** | | | **Implementation Outcomes** | |
| --- | --- | --- | --- | --- | --- | --- | --- | --- | --- | --- |
|  | **Cobb angle** | **ATR^a^** | **Spinal flexibility** | **Respiratory function** | **Postural control / Gait** | **Health-related quality of life** | **Pain** | **Body image** | **Adherence** | **Dropout/Loss-to-Follow-Up Rates** |
| **Real-Time Synchronous Therapist Supervision (n = 5)** | | | | | | | | | | |
| Andrade et al. [17] | ↓ |  |  |  |  |  |  |  |  |  |
| Dursun et al. [35] | ↑ | ○ |  | ↑ |  |  |  |  |  |  |
| Moraes et al. [41] |  |  | ↑ |  | ↑ |  |  |  |  |  |
| Ozger et al. [43] |  |  | ↑ | ↑ | ↑ | ↑ |  |  | ↑ |  |
| Chen et al. [45] | ↑ | ↑ |  |  |  | ↑ |  |  | ↑ | ↑ |
| **Total, n** | **3** | **2** | **2** | **2** | **2** | **2** | **–** | **–** | **2** | **1** |
| Favorable effect direction, n (%) | 2 (66.6%) | 1 (50.0%) | 2 (100.0%) | 2 (100.0%) | 2 (100.0%) | 2 (100.0%) | – | – | 2 (100.0%) | 1 (100.0%) |
| Unfavorable effect direction, n (%) | 1 (33.3%) | 0 (0.0%) | 0 (0.0%) | 0 (0.0%) | 0 (0.0%) | 0 (0.0%) | – | – | 0 (0.0%) | 0 (0.0%) |
| No clear effect direction, n (%) | 0 (0.0%) | 1 (50.0%) | 0 (0.0%) | 0 (0.0%) | 0 (0.0%) | 0 (0.0%) | – | – | 0 (0.0%) | 0 (0.0%) |
| **Asynchronous Technology-Mediated Remote Monitoring (n = 3)** | | | | | | | | | | |
| Kisa et al. [37] | ↑ | ↑ |  | ↑ |  |  | ↑ | ↑ | ○ | ○ |
| Lau et al. [38] | ○ |  |  |  |  | ↑ |  |  | ○ |  |
| Yuan et al. [44] | ↑ | ○ |  |  | ↑ |  |  |  | ↑ | ↑ |
| **Total, n** | **3** | **2** | **–** | **1** | **1** | **1** | **1** | **1** | **3** | **2** |
| Favorable effect direction, n (%) | 2 (66.7%) | 1 (50.0%) | – | 1 (100.0%) | 1 (100.0%) | 1 (100.0%) | 1 (100.0%) | 1 (100.0%) | 1 (33.3%) | 1 (50.0%) |
| Unfavorable effect direction, n (%) | 0 (0.0%) | 0 (0.0%) | – | 0 (0.0%) | 0 (0.0%) | 0 (0.0%) | 0 (0.0%) | 0 (0.0%) | 0 (0.0%) | 0 (0.0%) |
| No clear effect direction, n (%) | 1 (33.3%) | 1 (50.0%) | – | 0 (0.0%) | 0 (0.0%) | 0 (0.0%) | 0 (0.0%) | 0 (0.0%) | 2 (66.7%) | 1 (50.0%) |
| **Self-Directed Practice with Minimal Supervision (n = 3)** | | | | | | | | | | |
| Fishman [36] | ↑ |  |  |  |  |  |  |  | ↓ |  |
| Tombak et al. [39] |  | ○ |  |  |  | ○ |  | ○ | ○ |  |
| Zapata et al. [40] |  |  |  |  |  |  | ↓ |  | ↓ | ↓ |
| **Total, n** | **1** | **1** | **–** | **–** | **–** | **1** | **1** | **1** | **3** | **1** |
| Favorable effect direction, n (%) | 1 (100.0%) | 0 (0.0%) | – | – | – | 0 (0.0%) | 0 (0.0%) | 0 (0.0%) | 0 (0.0%) | 0 (0.0%) |
| Unfavorable effect direction, n (%) | 0 (0.0%) | 0 (0.0%) | – | – | – | 0 (0.0%) | 0 (0.0%) | 1 (100.0%) | 2 (66.7%) | 1 (100.0%) |
| No clear effect direction, n (%) | 0 (0.0%) | 1 (100.0%) | – | – | – | 1 (100.0%) | 1 (100.0%) | 0 (0.0%) | 1 (33.3%) | 0 (0.0%) |

↑ = favorable effect direction; ↓ = unfavorable effect direction; ○ = no clear effect direction. Blank cells indicate that the outcome was not assessed in that study. Direction of effect is reported regardless of statistical significance.

Studies were stratified into three supervision categories based on the mode and intensity of professional contact during the intervention. Real-time synchronous therapist supervision: the therapist actively observed and corrected exercise performance during each session, either in person or via live video link. Asynchronous technology-mediated remote monitoring: home-based practice enhanced by technology-enabled monitoring that permitted delayed or on-demand therapist intervention, without concurrent session supervision. Self-directed practice with minimal supervision: home-based practice following initial instruction, with professional contact restricted to periodic in-clinic visits or brief check-ins and no ongoing real-time or technology-mediated monitoring. The two feasibility studies (Wan et al. [34]; Nam et al. [42]) are excluded from this synthesis owing to the absence of a controlled comparator.

^a^ ATR: angle of trunk rotation.

Table S4. Direction-of-Effect Summary of Digital Health Interventions by Comparator Type.

| **Author [Ref]** | **Clinical Outcomes** | | | **Functional Outcomes** | | **Patient-Reported Outcomes** | | | **Implementation Outcomes** | |
| --- | --- | --- | --- | --- | --- | --- | --- | --- | --- | --- |
|  | **Cobb angle** | **ATR^a^** | **Spinal flexibility** | **Respiratory function** | **Postural control / Gait** | **Health-related quality of life** | **Pain** | **Body image** | **Adherence** | **Dropout/Loss-to-Follow-Up Rates** |
| **Inactive comparator (n = 2)** | | | | | | | | | | |
| Lau et al. [38] | ○ |  |  |  |  | ↑ |  |  | ○ |  |
| Fishman [36] | ↑ |  |  |  |  |  |  |  | ↓ |  |
| **Total, n** | **2** | **–** | **–** | **–** | **–** | **1** | **–** | **–** | **2** | **–** |
| Favorable effect direction, n (%) | 1 (50.0%) | – | – | – | – | 1 (100.0%) | – | – | 0 (0.0%) | – |
| Unfavorable effect direction, n (%) | 0 (0.0%) | – | – | – | – | 0 (0.0%) | – | – | 1 (50.0%) | – |
| No clear effect direction, n (%) | 1 (50.0%) | – | – | – | – | 0 (0.0%) | – | – | 1 (50.0%) | – |
| **Unsupervised Home Exercise (n = 3)** | | | | | | | | | | |
| Dursun et al. [35] | ↑ | ○ |  | ↑ |  |  |  |  |  |  |
| Ozger et al. [43] |  |  | ↑ | ↑ | ↑ | ↑ |  |  | ↑ |  |
| Chen et al. [45] | ↑ | ↑ |  |  |  | ↑ |  |  | ↑ | ↑ |
| **Total, n** | **2** | **2** | **1** | **2** | **1** | **2** | **–** | **–** | **2** | **1** |
| Favorable effect direction, n (%) | 2 (100.0%) | 1 (50.0%) | 1 (100.0%) | 2 (100.0%) | 1 (100.0%) | 2 (100.0%) | – | – | 2 (100.0%) | 1 (100.0%) |
| Unfavorable effect direction, n (%) | 0 (0.0%) | 0 (0.0%) | 0 (0.0%) | 0 (0.0%) | 0 (0.0%) | 0 (0.0%) | – | – | 0 (0.0%) | 0 (0.0%) |
| No clear effect direction, n (%) | 0 (0.0%) | 1 (50.0%) | 0 (0.0%) | 0 (0.0%) | 0 (0.0%) | 0 (0.0%) | – | – | 0 (0.0%) | 0 (0.0%) |
| **In-person Supervised Rehabilitation (n = 6)** | | | | | | | | | | |
| Andrade et al. [17] | ↓ |  |  |  |  |  |  |  |  |  |
| Kisa et al. [37] | ↑ | ↑ |  | ↑ |  |  | ↑ | ↑ | ○ | ○ |
| Tombak et al. [39] |  | ○ |  |  |  | ○ |  | ○ | ○ |  |
| Zapata et al. [40] |  |  |  |  |  |  | ↓ |  | ↓ | ↓ |
| Moraes et al. [41] |  |  | ↑ |  | ↑ |  |  |  |  |  |
| Yuan et al. [44] | ↑ | ○ |  |  | ↑ |  |  |  | ↑ | ↑ |
| **Total, n** | **3** | **3** | **1** | **1** | **2** | **1** | **2** | **2** | **4** | **3** |
| Favorable effect direction, n (%) | 2 (66.6%) | 1 (33.3%) | 1 (100.0%) | 1 (100.0%) | 2 (100.0%) | 0 (0.0%) | 1 (50.0%) | 1 (50.0%) | 1 (25.0%) | 1 (33.3%) |
| Unfavorable effect direction, n (%) | 1 (33.3%) | 0 (0.0%) | 0 (0.0%) | 0 (0.0%) | 0 (0.0%) | 0 (0.0%) | 1 (50.0%) | 0 (0.0%) | 1 (25.0%) | 1 (33.3%) |
| No clear effect direction, n (%) | 0 (0.0%) | 2 (66.7%) | 0 (0.0%) | 0 (0.0%) | 0 (0.0%) | 1 (100.0%) | 0 (0.0%) | 1 (50.0%) | 2 (50.0%) | 1 (33.3%) |

↑ = favorable effect direction; ↓ = unfavorable effect direction; ○ = no clear effect direction. Blank cells indicate that the outcome was not assessed in that study. Direction of effect is reported regardless of statistical significance. Studies were stratified into three comparator categories of increasing active treatment intensity: no-treatment control (usual care or absence of active intervention), unsupervised home exercise (home-based exercise without real-time professional supervision), and in-person supervised rehabilitation (face-to-face delivery by a qualified therapist). The two feasibility studies (Wan et al. [34]; Nam et al. [42]) are excluded from this synthesis owing to the absence of a controlled comparator.

^a^ ATR: angle of trunk rotation.

Table S5. Direction-of-Effect Summary of Digital Health Interventions by Intervention Dose.

| **Author [Ref]** | **Clinical Outcomes** | | | **Functional Outcomes** | | **Patient-Reported Outcomes** | | | **Implementation Outcomes** | |
| --- | --- | --- | --- | --- | --- | --- | --- | --- | --- | --- |
|  | **Cobb angle** | **ATR^a^** | **Spinal flexibility** | **Respiratory function** | **Postural control / Gait** | **Health-related quality of life** | **Pain** | **Body image** | **Adherence** | **Dropout/Loss-to-Follow-Up Rates** |
| **Short-term Intervention: Total Duration ≤12 Weeks (n = 5)** | | | | | | | | | | |
| Moraes et al. [41] |  |  | ↑ |  | ↑ |  |  |  |  |  |
| Ozger et al. [43] |  |  | ↑ | ↑ | ↑ | ↑ |  |  | ↑ |  |
| Zapata et al. [40] |  |  |  |  |  |  | ↓ |  | ↓ | ↓ |
| Dursun et al. [35] | ↑ | ○ |  | ↑ |  |  |  |  |  |  |
| Tombak et al. [39] |  | ○ |  |  |  | ○ |  | ○ | ○ |  |
| **Total, n** | **1** | **2** | **2** | **2** | **2** | **2** | **1** | **1** | **3** | **1** |
| Favorable effect direction, n (%) | 1 (100.0%) | 0 (0.0%) | 2 (100.0%) | 2 (100.0%) | 2 (100.0%) | 1 (50.0%) | 0 (0.0%) | 0 (0.0%) | 1 (33.3%) | 0 (0.0%) |
| Unfavorable effect direction, n (%) | 0 (0.0%) | 0 (0.0%) | 0 (0.0%) | 0 (0.0%) | 0 (0.0%) | 0 (0.0%) | 1 (100.0%) | 0 (0.0%) | 1 (33.3%) | 1 (100.0%) |
| No clear effect direction, n (%) | 0 (0.0%) | 2 (100.0%) | 0 (0.0%) | 0 (0.0%) | 0 (0.0%) | 1 (50.0%) | 0 (0.0%) | 1 (100.0%) | 1 (33.3%) | 0 (0.0%) |
| **Long-term Intervention: Total Duration >12 Weeks (n = 6)** | | | | | | | | | | |
| Fishman [36] | ↑ |  |  |  |  |  |  |  | ↓ |  |
| Andrade et al. [17] | ↓ |  |  |  |  |  |  |  |  |  |
| Kisa et al. [37] | ↑ | ↑ |  | ↑ |  |  | ↑ | ↑ | ○ | ○ |
| Lau et al. [38] | ○ |  |  |  |  | ↑ |  |  | ○ |  |
| Yuan et al. [44] | ↑ | ○ |  |  | ↑ |  |  |  | ↑ | ↑ |
| Chen et al. [45] | ↑ | ↑ |  |  |  | ↑ |  |  | ↑ | ↑ |
| **Total, n** | **6** | **3** | **–** | **1** | **1** | **2** | **1** | **1** | **5** | **3** |
| Favorable effect direction, n (%) | 4 (66.7%) | 2 (66.7%) | – | 1 (100.0%) | 1 (100.0%) | 2 (100.0%) | 1 (100.0%) | 1 (100.0%) | 2 (40.0%) | 2 (66.7%) |
| Unfavorable effect direction, n (%) | 1 (16.7%) | 0 (0.0%) | – | 0 (0.0%) | 0 (0.0%) | 0 (0.0%) | 0 (0.0%) | 0 (0.0%) | 1 (20.0%) | 0 (0.0%) |
| No clear effect direction, n (%) | 1 (16.7%) | 1 (33.3%) | – | 0 (0.0%) | 0 (0.0%) | 0 (0.0%) | 0 (0.0%) | 0 (0.0%) | 2 (40.0%) | 1 (33.3%) |

↑ = favorable effect direction; ↓ = unfavorable effect direction; ○ = no clear effect direction. Blank cells indicate that the outcome was not assessed in that study. Direction of effect is reported regardless of statistical significance. Studies were stratified into two subgroups based on total intervention duration: short-term intervention (total duration ≤12 weeks) and long-term intervention (total duration >12 weeks). The two feasibility studies (Wan et al. [34]; Nam et al. [42]) are excluded from this synthesis owing to the absence of a controlled comparator.

^a^ ATR: angle of trunk rotation.

Table S6. Direction-of-Effect Summary of Digital Health Interventions by Bracing Co-intervention Status.

| **Author [Ref]** | **Clinical Outcomes** | | | **Functional Outcomes** | | **Patient-Reported Outcomes** | | | **Implementation Outcomes** | |
| --- | --- | --- | --- | --- | --- | --- | --- | --- | --- | --- |
|  | **Cobb angle** | **ATR^a^** | **Spinal flexibility** | **Respiratory function** | **Postural control / Gait** | **Health-related quality of life** | **Pain** | **Body image** | **Adherence** | **Dropout/Loss-to-Follow-Up Rates** |
| **Combined with Bracing as Co-intervention (n = 3)** | | | | | | | | | | |
| Andrade et al. [17] | ↓ |  |  |  |  |  |  |  |  |  |
| Dursun et al. [35] | ↑ | ○ |  | ↑ |  |  |  |  |  |  |
| Chen et al. [45] | ↑ | ↑ |  |  |  | ↑ |  |  | ↑ | ↑ |
| **Total, n** | **3** | **2** | **–** | **1** | **–** | **1** | **–** | **–** | **1** | **1** |
| Favorable effect direction, n (%) | 2 (66.6%) | 1 (50.0%) | – | 1 (100.0%) | – | 1 (100.0%) | – | – | 1 (100.0%) | 1 (100.0%) |
| Unfavorable effect direction, n (%) | 1 (33.3%) | 0 (0.0%) | – | 0 (0.0%) | – | 0 (0.0%) | – | – | 0 (0.0%) | 0 (0.0%) |
| No clear effect direction, n (%) | 0 (0.0%) | 1 (50.0%) | – | 0 (0.0%) | – | 0 (0.0%) | – | – | 0 (0.0%) | 0 (0.0%) |
| **No Bracing Co-intervention (n = 8)** | | | | | | | | | | |
| Fishman [36] | ↑ |  |  |  |  |  |  |  | ↓ |  |
| Kisa et al. [37] | ↑ | ↑ |  | ↑ |  |  | ↑ | ↑ | ○ | ○ |
| Lau et al. [38] | ○ |  |  |  |  | ↑ |  |  | ○ |  |
| Tombak et al. [39] |  | ○ |  |  |  | ○ |  | ○ | ○ |  |
| Zapata et al. [40] |  |  |  |  |  |  | ↓ |  | ↓ | ↓ |
| Moraes et al. [41] |  |  | ↑ |  | ↑ |  |  |  |  |  |
| Ozger et al. [43] |  |  | ↑ | ↑ | ↑ | ↑ |  |  | ↑ |  |
| Yuan et al. [44] | ↑ | ○ |  |  | ↑ |  |  |  | ↑ | ↑ |
| **Total, n** | **4** | **3** | **2** | **2** | **3** | **3** | **2** | **2** | **7** | **3** |
| Favorable effect direction, n (%) | 3 (75.0%) | 1 (33.3%) | 2 (100.0%) | 2 (100.0%) | 3 (100.0%) | 2 (66.7%) | 1 (50.0%) | 1 (50.0%) | 2 (28.6%) | 1 (33.3%) |
| Unfavorable effect direction, n (%) | 0 (0.0%) | 0 (0.0%) | 0 (0.0%) | 0 (0.0%) | 0 (0.0%) | 0 (0.0%) | 1 (50.0%) | 0 (0.0%) | 2 (28.6%) | 1 (33.3%) |
| No clear effect direction, n (%) | 1 (25.0%) | 2 (66.7%) | 0 (0.0%) | 0 (0.0%) | 0 (0.0%) | 1 (33.3%) | 0 (0.0%) | 1 (50.0%) | 3 (42.9%) | 1 (33.3%) |

↑ = favorable effect direction; ↓ = unfavorable effect direction; ○ = no clear effect direction. Blank cells indicate that the outcome was not assessed in that study. Direction of effect is reported regardless of statistical significance. Studies were stratified into two categories based on whether bracing was a protocolized component of the trial: combined with bracing (bracing prescribed in both intervention and comparator arms) and no bracing co-intervention (bracing not prescribed in either arm). The two feasibility studies (Wan et al. [34]; Nam et al. [42]) are excluded from this synthesis owing to the absence of a controlled comparator.

^a^ ATR: angle of trunk rotation.

Table S7. Sensitivity Analysis: Direction-of-Effect Vote Counting Restricted to Studies at Low or Some Concerns Risk of Bias

| **Outcome** | **Primary synthesis (n = 11)** | | | | | **Sensitivity synthesis (n = 9)** | | | | | **Direction change** | **Robustness verdict** |
| --- | --- | --- | --- | --- | --- | --- | --- | --- | --- | --- | --- | --- |
|  | **n** | **↑** | **↓** | **○** | **% ↑** | **n** | **↑** | **↓** | **○** | **% ↑** |  |  |
| **Domain 1 — Clinical Outcomes** | | | | | | | | | | | | |
| Cobb angle | 7 | 5 | 1 | 1 | 71.4% | 5 | 4 | 1 | 0 | 80.0% | +8.6% | Robust |
| Angle of trunk rotation (ATR) | 5 | 2 | 0 | 3 | 40.0% | 5 | 2 | 0 | 3 | 40.0% | No change | Unchanged |
| Spinal flexibility | 2 | 2 | 0 | 0 | 100.0% | 2 | 2 | 0 | 0 | 100.0% | No change | Unchanged |
| **Domain 2 — Functional Outcomes** | | | | | | | | | | | | |
| Respiratory function | 3 | 3 | 0 | 0 | 100.0% | 3 | 3 | 0 | 0 | 100.0% | No change | Unchanged |
| Postural control / Gait | 3 | 3 | 0 | 0 | 100.0% | 3 | 3 | 0 | 0 | 100.0% | No change | Unchanged |
| **Domain 3 — Patient-Reported Outcomes** | | | | | | | | | | | | |
| Health-related quality of life | 4 | 3 | 0 | 1 | 75.0% | 3 | 2 | 0 | 1 | 66.7% | −8.3% | Largely robust |
| Pain | 2 | 1 | 1 | 0 | 50.0% | 2 | 1 | 1 | 0 | 50.0% | No change | Unchanged |
| Body image | 2 | 1 | 0 | 1 | 50.0% | 2 | 1 | 0 | 1 | 50.0% | No change | Unchanged |
| **Domain 4 — Implementation Outcomes** | | | | | | | | | | | | |
| Adherence | 8 | 3 | 2 | 3 | 37.5% | 6 | 3 | 1 | 2 | 50.0% | +12.5% | Signal improves |
| Dropout/Loss-to-Follow-Up Rates | 4 | 2 | 1 | 1 | 50.0% | 4 | 2 | 1 | 1 | 50.0% | No change | Unchanged |

Fishman [36]: excluded from sensitivity analysis (ROBINS-I: serious risk of bias).

Lau et al. [38]: excluded from sensitivity analysis (RoB 2: high risk of bias).

Robustness criteria: Robust or Signal improves, favorable proportion unchanged or increased after exclusion; Largely robust, attenuation ≤10% with favorable majority retained; Not robust, favorable signal disappears; Unchanged, neither excluded study contributed data to this outcome.

Table S8. Certainty of the evidence (GRADE) for each outcome in the direction-of-effect synthesis of digital health interventions for scoliosis rehabilitation.

| **Outcome** | **No. of studies (designs)** | **Direction of effect (↑/↓/○)** | **Risk of bias** | **Inconsistency** | **Indirectness** | **Imprecision** | **Certainty (GRADE)** |
| --- | --- | --- | --- | --- | --- | --- | --- |
| **Domain 1 — Clinical outcomes** | | | | | | | |
| Cobb angle | 7 (4 RCTs, 2 cohort studies, 1 non-randomized controlled trial) | ↑5, ↓1, ○1 (71.4%) | Not serious^a^ | Not serious | Not serious | Serious^b^ | Moderate (⊕⊕⊕◯) |
| Angle of trunk rotation | 5 (4 RCTs, 1 cohort study) | ↑2, ↓0, ○3 (40.0%) | Serious^c^ | Serious^d^ | Not serious | Serious | Very low (⊕◯◯◯) |
| Spinal flexibility | 2 (1 RCT, 1 cohort study) | ↑2, ↓0, ○0 (100%) | Not serious | Not serious | Not serious | Very serious^e^ | Low (⊕⊕◯◯) |
| **Domain 2 — Functional outcomes** | | | | | | | |
| Respiratory function | 3 (3 RCTs) | ↑3, ↓0, ○0 (100%) | Serious | Not serious | Not serious | Serious | Low (⊕⊕◯◯) |
| Postural control and gait | 3 (2 RCTs, 1 cohort study) | ↑3, ↓0, ○0 (100%) | Not serious | Not serious | Serious^f^ | Serious | Low (⊕⊕◯◯) |
| **Domain 3 — Patient-reported outcomes** | | | | | | | |
| Health-related quality of life | 4 (3 RCTs, 1 cohort study) | ↑3, ↓0, ○1 (75.0%) | Serious^g^ | Not serious | Not serious | Serious | Low (⊕⊕◯◯) |
| Pain | 2 (2 RCTs) | ↑1, ↓1, ○0 (50.0%) | Serious | Serious | Not serious | Serious | Very low (⊕◯◯◯) |
| Body image | 2 (2 RCTs) | ↑1, ↓0, ○1 (50.0%) | Serious | Serious | Not serious | Serious | Very low (⊕◯◯◯) |
| **Domain 4 — Implementation outcomes** | | | | | | | |
| Adherence | 8 (6 RCTs, 1 cohort study, 1 non-randomized controlled trial) | ↑3, ↓2, ○3 (37.5%) | Serious^h^ | Serious | Not serious | Serious | Very low (⊕◯◯◯) |
| Dropout/Loss-to-Follow-Up Rates | 4 (3 RCTs, 1 cohort study) | ↑2, ↓1, ○1 (50.0%) | Serious | Serious | Not serious | Serious | Very low (⊕◯◯◯) |

All outcome-level bodies of evidence included randomized controlled trials and therefore started at high certainty before being rated down across the four GRADE domains. Publication bias was not used for downgrading, and rating-up criteria were not applicable to direction-of-effect data. Where the same reason for downgrading applied to more than one outcome, the explanatory footnote is given at its first occurrence.

Certainty: ⊕⊕⊕⊕, high; ⊕⊕⊕◯, moderate; ⊕⊕◯◯, low; ⊕◯◯◯, very low.

Direction of effect: ↑, number of studies favoring the digital intervention; ↓, number favoring the comparator; ○, number with no clear direction; the percentage is the proportion of studies favoring the digital intervention.

^a^ Not downgraded for risk of bias; the favorable signal strengthened in the sensitivity analysis (71.4% to 80.0%).

^b^ Downgraded for imprecision: no pooled estimate, with few and small contributing studies.

^c^ Downgraded for risk of bias: contributing randomized trials had some concerns.

^d^ Downgraded for inconsistency: the direction of effect was inconsistent across studies.

^e^ Downgraded by two levels for imprecision: only two small studies were available.

^f^ Downgraded for indirectness: outcomes were surrogate functional measures.

^g^ Downgraded for risk of bias: the favorable direction was partly driven by a high-risk study and attenuated in the sensitivity analysis.

^h^ Downgraded for risk of bias: contributing studies included a non-randomized study at serious risk and a trial at high risk, and reporting of adherence monitoring was incomplete (TIDieR).

GRADE: Grading of Recommendations Assessment, Development and Evaluation; RCT: randomized controlled trial.

References:

17. Mantelatto Andrade R, Gomes Santana B, Verttú Schmidt A, et al. Effect of traditional rehabilitation programme versus telerehabilitation in adolescents with idiopathic scoliosis during the COVID-19 pandemic: a cohort study. J Rehabil Med. Feb 26, 2024;56:jrm5343. [doi: 10.2340/jrm.v56.5343] [Medline: 38407430]

34. Wan FKW, Mak ATH, Chung CWY, Yip JYW. Development of a motion-based video game for postural training: a feasibility study on older adults with adult degenerative scoliosis. IEEE Trans Neural Syst Rehabil Eng. 2024;32:2106-2113. [doi: 10.1109/TNSRE.2024.3398029] [Medline: 38717877]

35. Manzak Dursun AS, Ozyilmaz S, Ucgun H, Elmadag NM. The effect of pilates-based exercise applied with hybrid telerehabilitation method in children with adolescent idiopathic scoliosis: a randomized clinical trial. Eur J Pediatr. Feb 2024;183(2):759-767. [doi: 10.1007/s00431-023-05340-2] [Medline: 37993666]

36. Fishman LM. Isometric yoga-like maneuvers improve adolescent idiopathic scoliosis-a nonrandomized control trial. Glob Adv Health Med. 2021;10:2164956120988259. [doi: 10.1177/2164956120988259] [Medline: 33717658]

37. Kisa EP, Tarakcı E, Leblebici G, Ozdincler AR, Kasapcopur O. Exercise programs for scoliosis in juvenile idiopathic arthritis: a randomized-controlled study. Rheumatol Int. Mar 2024;44(3):441-449. [doi: 10.1007/s00296-023-05439-7] [Medline: 37668835]

38. Lau RWL, Cheuk KY, Ng BKW, et al. Effects of a home-based exercise intervention (E-Fit) on bone density, muscle function, and quality of life in girls with adolescent idiopathic scoliosis (AIS): a pilot randomized controlled trial. Int J Environ Res Public Health. Oct 17, 2021;18(20):10899. [doi: 10.3390/ijerph182010899] [Medline: 34682668]

39. Tombak K, Yüksel İ, Ozsoy U, Yıldırım Y, Karaşin S. A comparison of the effects of supervised versus home schroth exercise programs with adolescent idiopathic scoliosis. Children (Basel). Mar 17, 2024;11(3):354. [doi: 10.3390/children11030354] [Medline: 38539389]

40. Zapata KA, Wang-Price SS, Sucato DJ, Thompson M, Trudelle-Jackson E, Lovelace-Chandler V. Spinal stabilization exercise effectiveness for low back pain in adolescent idiopathic scoliosis: a randomized trial. Pediatr Phys Ther. 2015;27(4):396-402. [doi: 10.1097/PEP.0000000000000174] [Medline: 26397085]

41. Moraes ÍA, Cardoso A, Soares AB, de Oliveira DAR, Júnior EL. Combining immersive virtual reality and postural therapy for scoliosis treatment and prevention in children. Res Biomed Eng. 2022;38(3):1003-1016. [doi: 10.1007/s42600-022-00235-x]

42. Nam YG, Park SY, Kwon BS. Rehabilitation exercise using a smart-bar device with augmented reality guide function. Int J Precis Eng Manuf. Jul 2023;24(7):1263-1270. [doi: 10.1007/s12541-023-00815-6]

43. Ozger EY, Mustafaoglu R, Nimetoglu B, Akgul T. Efficacy of virtual reality-based rehabilitation following posterior fusion in adolescent idiopathic scoliosis: a single-blind randomized trial. Eur Spine J. Jan 2026;35(1):36-45. [doi: 10.1007/s00586-025-09501-9] [Medline: 41201608]

44. Yuan W, Shi W, Chen L, et al. Digital physiotherapeutic scoliosis-specific exercises for adolescent idiopathic scoliosis: a randomized clinical trial. JAMA Netw Open. Feb 3, 2025;8(2):e2459929. [doi: 10.1001/jamanetworkopen.2024.59929] [Medline: 39964686]

45. Chen T, Zhou H, Chen Q, et al. Effects of bracing combined with tele-rehabilitation-guided family physiotherapeutic scoliosis-specific exercises on adolescent idiopathic scoliosis. Orthop Surg. Mar 2026;18(3):511-522. [doi: 10.1111/os.70265] [Medline: 41615191]
